# Change, stability, and instability in the Pavlovian guidance of behaviour from adolescence to young adulthood

**DOI:** 10.1371/journal.pcbi.1006679

**Published:** 2018-12-31

**Authors:** Michael Moutoussis, Edward T. Bullmore, Ian M. Goodyer, Peter Fonagy, Peter B. Jones, Raymond J. Dolan, Peter Dayan, on behalf of The Neuroscience in Psychiatry Network Research Consortium

**Affiliations:** 1 Wellcome Centre for Human Neuroimaging, University College London, London, United Kingdom; 2 Max Planck Centre for Computational Psychiatry and Ageing, University College London, United Kingdom; 3 Department of Psychiatry, University of Cambridge, Cambridge, United Kingdom; 4 Cambridgeshire and Peterborough National Health Service Foundation Trust, Cambridge, United Kingdom; 5 Medical Research Council/Wellcome Trust Behavioural and Clinical Neuroscience Institute, University of Cambridge, Cambridge, United Kingdom; 6 ImmunoPsychiatry, GlaxoSmithKline Research and Development, Stevenage, United Kingdom; 7 Research Department of Clinical, Educational and Health Psychology, University College London, London, United Kingdom; 8 Max Planck Institute of Biological Cybernetics, Tübingen, Germany; Harvard University, UNITED STATES

## Abstract

Pavlovian influences are important in guiding decision-making across health and psychopathology. There is an increasing interest in using concise computational tasks to parametrise such influences in large populations, and especially to track their evolution during development and changes in mental health. However, the developmental course of Pavlovian influences is uncertain, a problem compounded by the unclear psychometric properties of the relevant measurements. We assessed Pavlovian influences in a longitudinal sample using a well characterised and widely used Go-NoGo task. We hypothesized that the strength of Pavlovian influences and other ‘psychomarkers’ guiding decision-making would behave like traits. As reliance on Pavlovian influence is not as profitable as precise instrumental decision-making in this Go-NoGo task, we expected this influence to decrease with higher IQ and age. Additionally, we hypothesized it would correlate with expressions of psychopathology. We found that Pavlovian effects had weak temporal stability, while model-fit was more stable. In terms of external validity, Pavlovian effects decreased with increasing IQ and experience within the task, in line with normative expectations. However, Pavlovian effects were poorly correlated with age or psychopathology. Thus, although this computational construct did correlate with important aspects of development, it does not meet conventional requirements for tracking individual development. We suggest measures that might improve psychometric properties of task-derived Pavlovian measures for future studies.

## Author summary

Choice behaviour is guided by Pavlovian influences, so that particular features of a situation, e.g. if one seeks to gain rewards to avoid losses, privilege certain decisions over others–here, to be active versus vs. inactive respectively. Such privileging may be useful but may also impair optimal instrumental behaviour. We examined the balance of Pavlovian and instrumental guidance of choice in healthy, 14-24-year-old participants and found that young people with higher IQ relied less on Pavlovian guidance. Experience with a task changed Pavlovian guidance in a rational manner, so that it was used less when unnecessary. On the other hand, the degree of Pavlovian influence was not a highly stable trait and did not depend on age or mood. The degree of unpredictability in choice emerged as a crucial individual characteristic, associated with stronger Pavlovian influences yet more stable than them as a disposition.

## Introduction

A leitmotif in the nascent field of computational psychiatry [1–4] is that carefully curated cognitive tasks can be used to identify latent dimensions of decision-making. These parametrize process accounting for how the tasks are solved, and are identified according to the models that best fit behaviour. Individuals are characterized according to their coordinates in these dimensions and it is by this means that dysfunction is delineated. A number of such dimensions, quantifying features such as reward and punishment sensitivity [5,6], uncertainty [7,8], exploration [9], metacognition [10], interpersonal modelling [11] have been extensively investigated in laboratory tasks.

However, to characterize individuals in a psychometrically competent manner, it does not suffice to have external validity in terms of indices of development and pathology. Temporal stability is also crucial [12,13].Stability and related psychometric properties are increasingly important as computational psychiatry moves from describing differences between selected groups of individuals, for example well vs. ill groups, to describing individual change attributable to development, vulnerability to psychopathology, and recovery from psychiatric disorder. Stability is also crucial if computational parameters are to guide diagnosis and personalized psychiatry. Unstable measures may have predictive value [14,15], especially if their variability can be understood [16], but cannot easily characterise individual trajectories. It is unclear whether computational tasks that have been well validated in the laboratory, and which are starting to be used in epidemiological samples studies [17,18], have psychometric properties sufficient to pinpoint individual dispositions. In particular, while learning tasks are amongst the most popular in computational psychiatry, it is not clear if they bear repetition, for instance whether the identity of the best fitting process model remains the same when they are applied again. Furthermore, we often do not know if parameters inferred by using these best models are psychometrically reliable, covarying with traits, or change with the individual’s state and experience.

Here, we study the population distribution and psychometrics of a paradigmatic computational measure, namely the extent to which an individual’s decision-making is guided by Pavlovian influences [19]. This is the direct predisposition to prefer particular actions in response to features of a stimulus, such as the appetitive or aversive consequences that it predicts. This predisposition can help or hinder instrumental behaviour, which is defined in terms of the contingency between action and outcome. Pavlovian biases have often been studied because of their translational relevance for anxiety, post-traumatic stress, and other disorders [20–23]. The incidence of psychiatric symptoms where Pavlovian influences have been implicated rises in adolescence. For example, we recently described a peak in mood symptoms in a large non-clinical sample around the age of 16 in females [13]. It is thus important to examine how Pavlovian influences vary with age as well as characteristics such as mood, sex and IQ [1]. This in turn makes psychometric questions related to tasks assessing Pavlovian phenomena particularly pressing.

Pavlovian influences elicited by predictions of reward and punishment have been extensively studied through variants of a Go-NoGo task [19,24–26]. Here, subjects prefer to execute, rather than withhold, actions in proportion to their expectations of winning money. This Pavlovian ‘bias’ is quantified as a perturbation of a standard reinforcement learning model, using a form of bonus that is proportional to the predicted value associated with the stimuli concerned [25]. This rewards ‘Go’ actions in the face of ‘potential win’ stimuli and ‘No-Go’ actions in the face of ‘potential loss’ stimuli.

To assess Pavlovian bias and associated decision-making characteristics, we used an orthogonalised Go-NoGo task, wherein optimal decisions (Go vs. NoGo) are independent of the goal outcome (winning vs. avoiding loss). This has been extensively validated to assess Pavlovian bias, while much is known about neural function in this task [19,24,25,27]. We administered it on two occasions (termed ‘baseline’ and ‘long follow-up’) in a large, naturalistic, epidemiologically informed sample of 14 to 24 year olds [28]. We first validated the class of reinforcement-learning models developed in the laboratory in this population. In the process, we asked which model best described behaviour and ascertained that estimates of Pavlovian bias were robust with respect to secondary modelling details. We considered a model with differential sensitivity to wins and losses, which Guitart-Masip et al, 2014, found to fit behaviour best (‘valenced-sensitivity’ model). We also considered variants, particularly equal sensitivity to wins and losses, but differential learning to these outcomes (‘valenced-learning model’). We then compared the psychometric properties of the best models using real but also simulated data. We examined the external validity of the best models by assessing correlations between parameter values and the variables of age, IQ and mood. Calendar age is a key variable in development, albeit not the same as developmental time [29–31]. IQ is also an important yardstick, as theoretical [32] and experimental [33] findings motivate further examination of its relationship with Pavlovian tendencies. Specifically, we argued that in the orthogonalized Go-NoGo task used here, efficient instrumental learning rather than reliance on non-instrumental Pavlovian biases is most profitable. Therefore, participants with higher IQ might be expected to rely less on Pavlovian guidance. Finally, we used the ‘Mood and Feelings Questionnaire’, or MFQ for external validation. This was motivated by two considerations: first, research has suggested links between Pavlovian bias and so-called internalizing disorders [20,21,26]. Second, we have found that MFQ is a good simple proxy for the ‘general psychopathology factor’ (see S1 Fig and [13]). It may thus shed light into how Pavlovian bias contributes to psychiatric vulnerability or resilience in general, although further research should address associations with other, specific dimensions of psychopathology.

We examined longitudinal changes and correlations, particularly concerning the best fitting model and the trajectory of Pavlovian parameters. First, we performed a ‘short follow-up’ study over an interval of 6 months. This is short in developmental but not in test-retest terms. It helped us to subsequently interpret the results of our main, ‘long follow-up’ study, about 18 months post-baseline. Over and above model parameters, model-fit was of over-arching importance, as it assessed how well a specific cognitive model captures individual behaviour. We then explored the dependence of model-fit on age, task repetition and IQ.

We tested three hypotheses for the trajectory of the Pavlovian parameter. One is that the bias characterising an individual was a trait that remained stable over the time of our study. Alternatively, it might reflect a slowly changing developmental disposition, specifically one where younger participants were more strongly guided by Pavlovian biases. A third possibility is that Pavlovian biases reflected prior beliefs not only dependent on the context of ‘opportunity’ (appetitive trials) or ‘threat’ (aversive trials), but also dependent on other features of the context, such as ‘task taking place in a particular laboratory’. In the latter case, participants could update their prior beliefs about a link between appetitiveness and the appropriate action across test sessions.

## Results

Key descriptive characteristics of participants at baseline are shown in Table 1.

**Table 1:** Age, sex, ethnicity, mood and WASI total IQ distributions.

Age at baseline	Age at short follow-up	Age at long follow-up	Mood at baseline (MFQ)	IQ (WASI) at baseline	Sex	Ethnicity (UK convention)
Mean: 18.95	Mean: 19.38	Mean: 20.29	Mean: 18.39	Mean: 110.4	Female: 426	Asian: 70
Quartile 1: 16.45	Quartile 1: 17.39	Quartile 1: 17.69	Quartile 1: 8.00	Quartile 1: 103.0	Male: 392	Black: 34
Quartile 3: 21.23	Quartile 3: 21.30	Quartile 3: 22.51	Quartile 3: 24.00	Quartile 3: 118.0		Mixed: 54
Range: 14.10–24.00	Range: 14.93–24.91	Range: 15.11–26.49				Other: 18
						White: 591
						Undisclosed: 51

### Short time interval follow-up study

**Fig 1:**
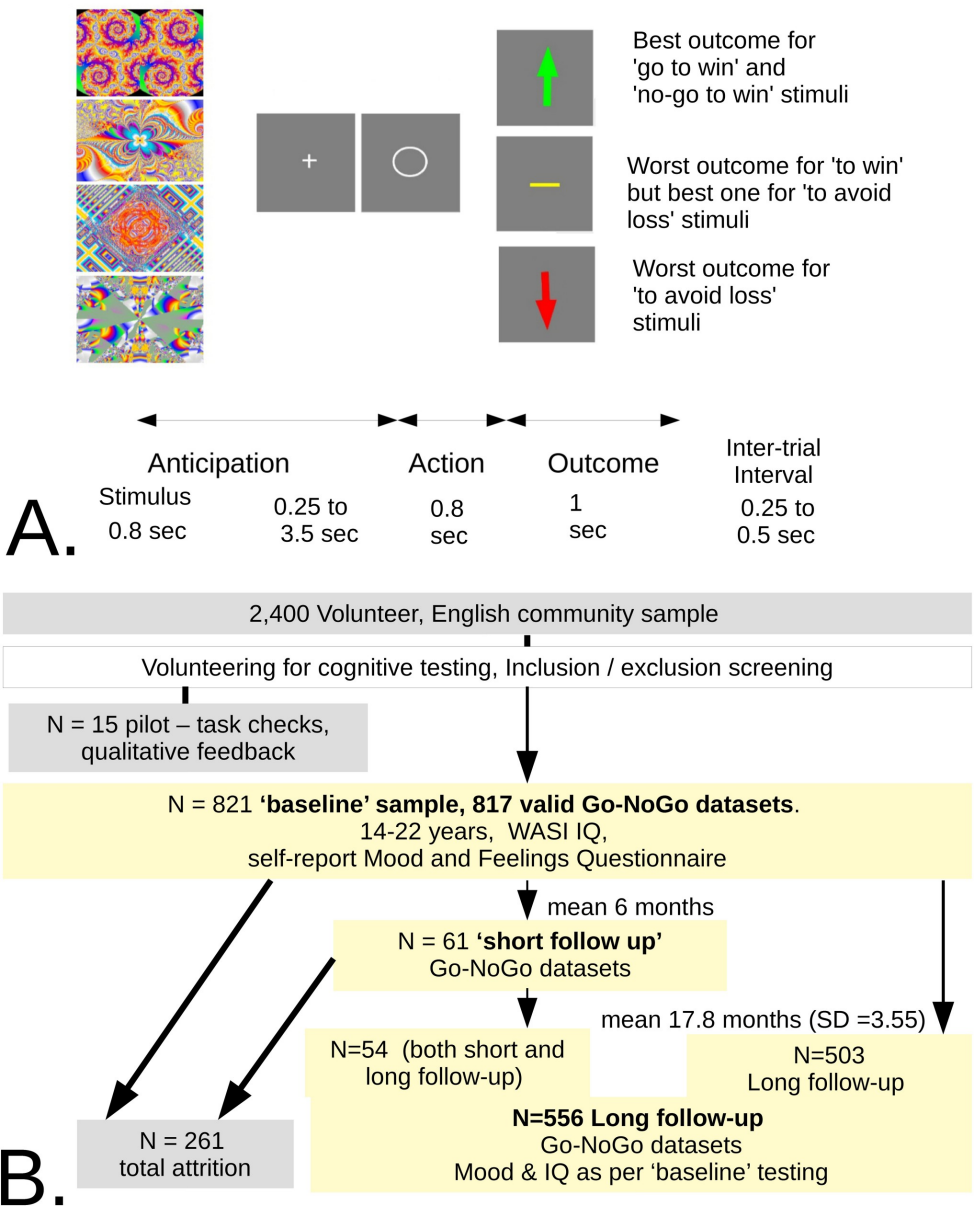
Study task and longitudinal structure. A. Go-NoGo task. Modified with permission from (26). Participants are presented with four stimuli for 36 trials each in fully randomized order. After a short ‘wait’ interval, they implement a decision of either to press or not press a button. Subjects discover by trial and error the two possible outcomes for choice of each stimulus (win/nothing or nothing/loss) and across trials learn which decision, ‘Go’ or ‘NoGo’, most often (with probability 0.8) leads to the best outcome. B. Longitudinal study structure, summarizing and illustrating the stages described in Methods.

We tested 61 participants at baseline, and then after an interval of 6 months (Fig 1), using the task briefly described above (and fully in Methods; very similar to [26]). We thus first explored temporal individual stability and group-level change over a time scale which was short in developmental terms. We report uncorrected Pearson *r* for approximately Gaussian quantities and Spearman *ρ* for non-Gaussian ones, using parameters inferred from the preferred model variant that emerged from model-comparisons. This was the ‘valenced learning’ variant, quite similar but not identical to the established one [19,24]. Please see the Methods section for details.

**Fig 2:**
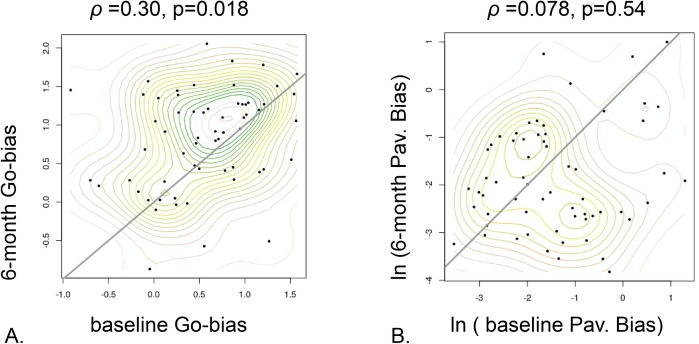
Parameter time dependency for the short follow-up study, baseline vs. 6 months later. **A.** Go Bias **B.** (log) Pavlovian bias. Statistically significant correlation is observed for the Go-bias but not for the Pavlovian bias.

For the overall propensity to choose action over inaction, parametrized by the ‘Go Bias’, baseline estimates were significantly correlated with short follow-up estimates, as hypothesized (*r =* 0.30, *p =* 0.018; Fig 2). However, this was not true for the other parameters. The Pavlovian bias, parametrizing the propensity to action in the context of opportunity and inaction in a context of loss, had *p =* 0.54. The motivational exchange rate, which measures how strongly likelihood of a choice depends on its value, had *p* = 0.55. For the learning rates for the appetitive and aversive contexts, *p* was 0.13, and 0.52 respectively. The irreducible noise parameter, quantifying decision variability that could not be reduced by learning motivating actions, had r = 0.24, p = 0.052. The extent to which the model accounted for behaviour, the integrated likelihood measure, was the most inter-correlated variable between baseline and short follow-up, *r =* 0.43, *p =* 0.00047. As we shall see below, similar results obtained in the larger, long follow-up study, suggesting that developmentally, individuals largely maintain their rank within the cohort with respect to this measure.

Next, we tested whether each of the parameters increased or decreased with task repetition. As we had no a priori hypotheses, we applied a Bonferroni correction for 6 tests, so that a corrected threshold of *p* = 0.05 corresponded to uncorrected *p* = 0.0083. We found that outcome sensitivity clearly increased (uncorrected Wilcoxon *p =* 5.5e-7) over the 6-month interval. There was weak evidence that Pavlovian bias decreased (from a median on 0.20 to 0.12; uncorr. t-test *p* = 0.024).

There was no significant change in the other parameters over the short follow-up, but there was good evidence that the median integrated likelihood increased (from -66.4 to -53.7, uncor. Wilc. *p* = 0.0038). 54 of the short-interval participants were also included in the long follow-up sample (Fig 1). They are included in the long follow-up analyses below, but their exclusion results in minimal change. For example, the baseline vs. long follow-up correlation *ρ* of Pavlovian bias does not change, while its p-value would drop slightly from 0.017 to 0.020.

Key results from the short-follow up study thus were that model fit was longitudinally the most stable measure, while the group shifted its outcome sensitivity and Pavlovian bias in the direction of benefitting performance, and it improved its model-fit.

### Large naturalistic study

**Fig 3:**
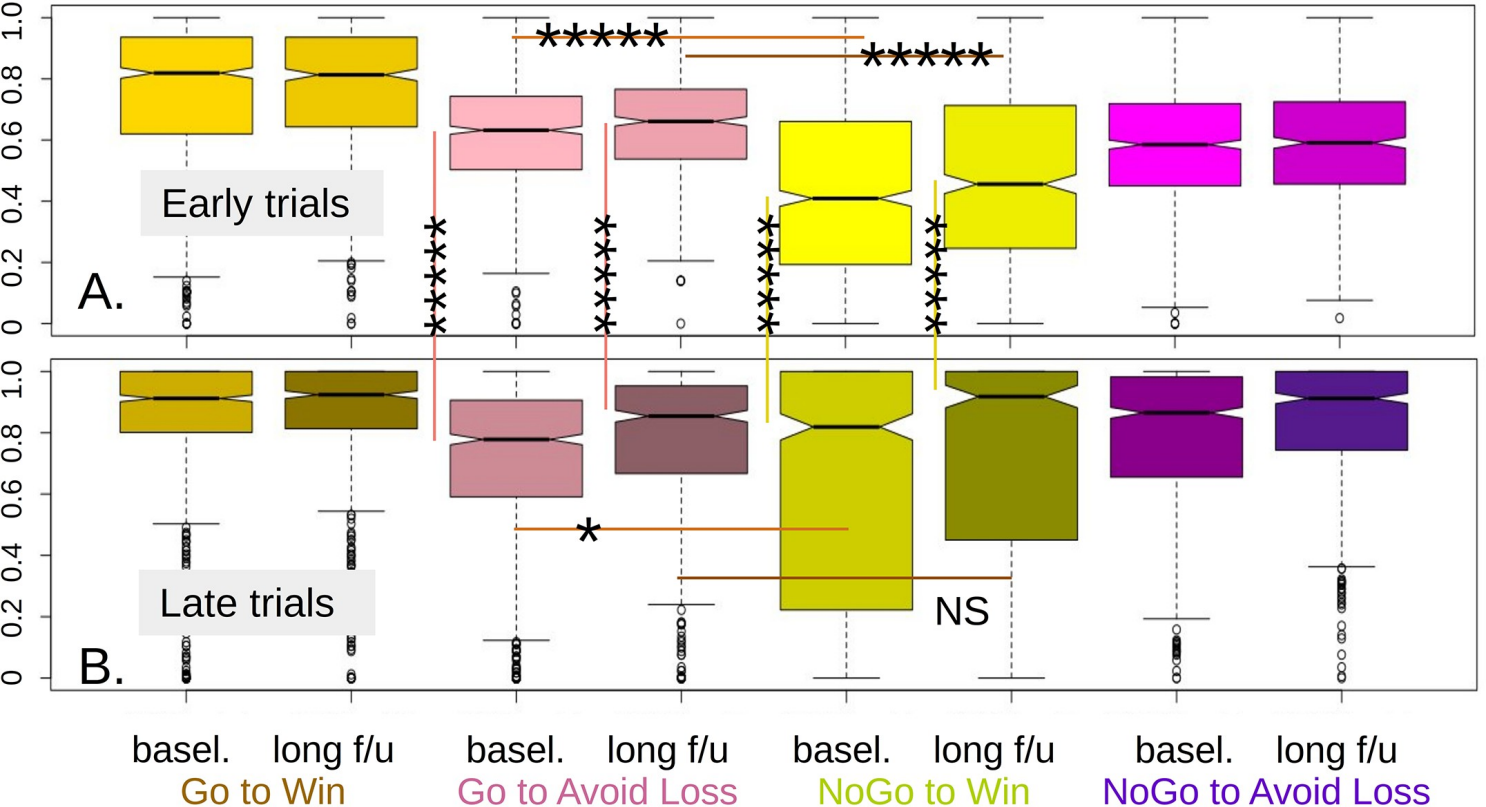
Raw performance in the all conditions. The middle two quartiles are in solid color, with the ‘avoid loss’ conditions in pink/purple and ‘win’ in gold/yellow. Just non-overlapping notches represent p = 0.05 for the uncorrected difference between two medians. One star denotes *p* <0.05 corrected for 8 comparisons; Five stars denote *p*_cor_ < 1e-10. Long follow-up is shown next to baseline. **A.** Performance weighted towards early trials, the weighing decreasing linearly to 0 for the middle of the task. No-Go to win (NG2W) showed median success rate slightly below chance, in line with the literature. **B.** Late trials. Performance reaches maximum for at least a quarter of participants in both appetitive conditions, but a quarter (long follow-up) or more (baseline) participants still perform below chance in NG2W. Long follow-up shows better performance than baseline in all except the easy Go-to-Win (G2W) conditions.

In the large naturalistic study, we first collected data from N_1_ = 817 participants (‘baseline’ sample). Of these, N_2_ = 556 (68%) also provided valid data at a follow-up session, on average 18 months later (‘long follow up’ sample). We first analysed performance simply in terms of the proportion of correct responses that participants achieved in each task condition, time during the task and session (Fig 3).

The characteristic ‘Pavlovian bias’ interaction pattern was seen, with Pavlovian-incongruent conditions showing worse performance than the corresponding congruent ones (Go to Win > NoGo to Win, NoGo to Avoid Loss > Go to Avoid Loss) at all stages. As shown in Fig 3A (‘early trials’ panel, second vs. third pair of boxes), G2AL showed a clearly better level starting level of performance than the other pavlovian-incongruent condition, NG2W (baseline difference: 18%, *p*_cor_ < 1e-10; long follow-up difference: 16%, *p*_cor_ < 1e-10). However, for the ‘late’ trials (Fig 3B) the improvement in median fraction of correct responses in G2AL was modest compared to those of NG2W. Hence, median performance in the latter now matched the former (baseline difference: -0.6%, *p*_cor_ < 0.05; long follow-up difference: -0.6%, *p*_cor_ = NS). This pattern suggests that not only Pavlovian congruency, but action and/or valence biases in learning and decision making need to be considered.

**Fig 4:**
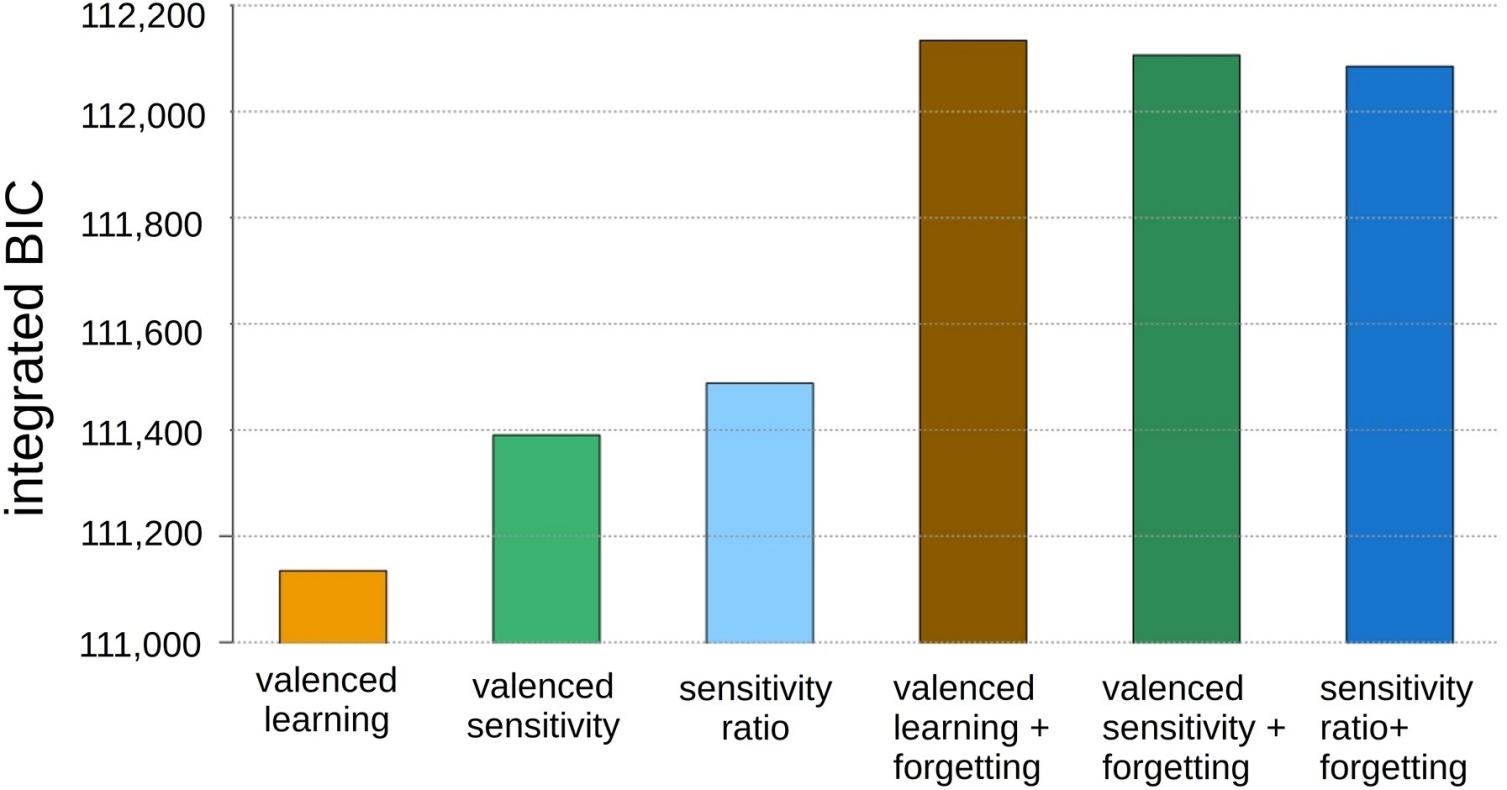
Comparison of fit quality for 6-parameter (left three) and 7-parameter (right three) models, using the baseline data. The ‘valenced learning’ (leftmost) model performs best, i.e. had the lowest integrated Bayesian Information Criterion score. Adding forgetting parameters (right three bars) worsened the fits due to the complexity penalties involved.

As descriptive statistics do not distinguish clearly the roles of Pavlovian bias and other processes of interest, we fitted a range of computational models capable of these distinctions to the data. We used the integrated likelihood (iL) and Bayesian Information Criterion (iBIC) to quantify complexity-corrected accuracy (Fig 4) and thereby compare models [24]. We assessed whether the identity of the best-fitting model remained the same over testing sessions, and whether estimates of Pavlovian bias were robust to secondary modelling considerations. We expected correlations for each task parameter across time to be positive. Accordingly, we report uncorrected *p*’s for Spearman correlations and Wilcoxon paired t-tests. We were also interested in the direction of any systematic change. Here, in the absence of a priori hypotheses, we report Wilcoxon tests, applying a Bonferroni correction for as many comparisons as there were parameters.

In the baseline sample, the valenced-learning model performed best (Fig 4, leftmost), with the valenced-sensitivity model 255.7 BIC units behind. Importantly, these two models produced very similar estimates for the parameters of interest here (r = 0.88 between models for the Pavlovian parameters, r = 0.93 for Go-bias, *p* <<1e-05). A variant of the valenced-senstivity model, the ‘sensitivity ratio’ model, also furnished highly similar Pavlovian bias estimates (e.g. r = 0.93, *p* <<1e-05 with valenced-learning). As a quality check, the ‘irreducible noise’ parameters, which quantify lack of attention and motivation-independent lapses, were reassuringly low (5–7%). In the long follow-up sample the valenced-learning model again obtained an advantage, here of 275.6 BIC units over the second-best, valenced-sensitivity model.

However, the mean advantage of the winning model per participant was only 0.15 BIC units at baseline, while the typical individual uncertainty in iBIC, estimated as the SD of BIC scores refitted to data generated using the exact mean population value of each parameter for the winning model, was 7.9 units. Thus, while evidence of >250 BIC units is considered overwhelming by conventional standards [34], we asked if in studies with N>500 it might arise by chance and, as importantly, what difference in predictive power it signifies. Using a paired Wilcoxon test to compare fits for the two models for the data obtained in the first (baseline) testing session gave *p* = 0.09, while for the long follow-up Wilcoxon *p* was 1.7e-4, overall providing evidence against a false positive finding. Similarly, using integrated likelihood estimates at the individual level yielded values of approximate protected exceedance probabilities of 0.569 for baseline and 0.974 for long follow-up in favour of valenced-learning [35]. However, when we asked if having a greater likelihood for the one model at baseline implied a similar ordering at long follow-up, a chi-square test showed no evidence (*p* = 0.38). Numerical studies later showed that even if individuals’ employment of a particular model [valenced learning or sensitivity] remained fixed, and so constituted a ‘type’, our relatively brief experiment would be under-powered to allocate individuals to their type reliably (see S1 Appendix, ‘In silico simulated agents’ reliability and biases’).

**Fig 5:**
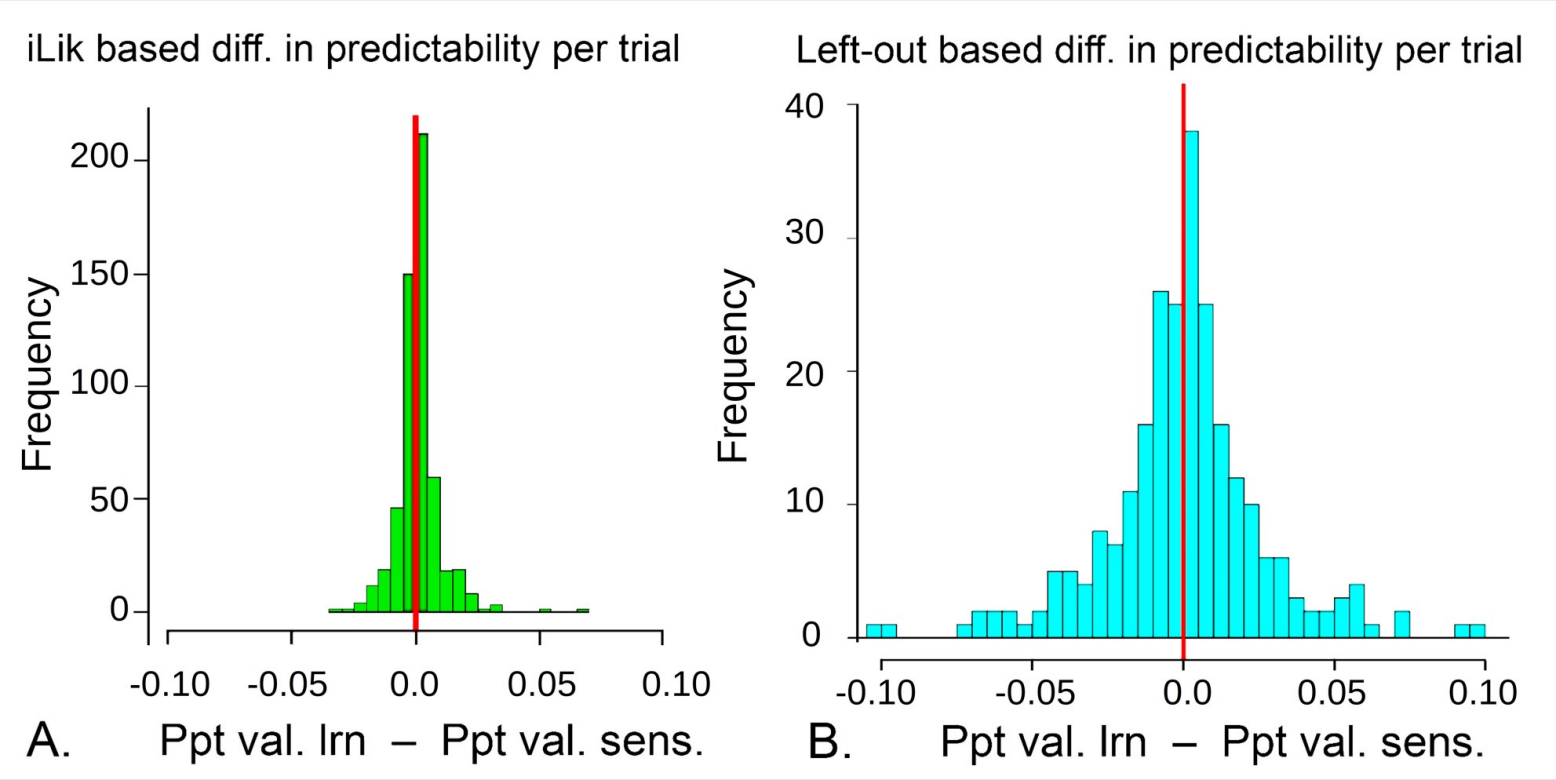
Model comparison based on Mean Prediction probability per trial (Ppt) in the long follow-up sample, showing that the difference in Ppt between the two best models is similar if one uses model-fitting vs. out-of-sample based methods **A.** ΔPpt estimated through a model fit measure, namely mean integrated likelihood per trial, N_2_ = 556. Both models have mean Ppt about 0.64. **B.** ΔPpt estimated by out-of-sample prediction of the 48^th^ and 96^th^ trials for each participant on a test subsample of N = 255. This out-of-sample comparison is more variable, but the resampling-based 95% confidence interval (CI) of the median difference is -0.0012 to 0.0026, consistent with A. If it were desirable to further reduce this CI, the estimate could be averaged over rotated out-of-sample trials, at the very considerable computational cost of re-estimating the entire model fit for each left-out sample.

We then estimated predictive power. First, we expressed the difference in BIC in terms of the probability of the model better predicting a participant’s decision per trial. Even at long follow-up, this difference was very small, mean ΔPpt = 0.0011, compared to a grand mean prediction probability per trial, Ppt = 0.64. We also introduced a new out-of-sample, or ‘left out likelihood’ (LOL) comparison method, suitable for tasks involving learning which present challenges for predictive tests. This is important, as predictive tests do not rely on the approximations inherent in the BIC and become more and more powerful as datasets become larger (interested readers are referred to the Methods section for validation and details). LOL testing confirmed in an unbiased manner that the likely difference in predictive power between the two best models was very small, interquartile range of ΔPpt -0.012 to 0.0026. Here the mean predictability per trial was 0.73 (see Fig 5 and Methods).

To further assess model quality, we examined correlations between parameters within each of the best models. The ones remaining significant after correction for multiple comparisons for the valenced-learning model are shown in Table 2 (valenced-sensitivity is similar). Four within-model significant correlations were consistently found at baseline and long follow-up. The Pavlovian bias was anticorrelated with both learning rates, while the motivational exchange rate correlated with the Go-bias and anticorrelated with irreducible noise. The motivational exchange rate is also known as reward sensitivity or inverse decision temperature. It can be seen as the power that a unit of additional reward (or loss) has to shift behaviour off indifference between choices. The correlations were also significant in the long follow-up sample (Pav. bias vs. appetitive and aversive learning rates: *ρ* = -0.14, -0.19, *p =* 0.016, 0.00011; motiv. Exchange rate vs. irreducible noise and Go bias: *ρ* = -0.13,0.14, *p =* 0.043, 0.020). S2 Table shows similar results for the valenced sensitivity model.

**Table 2:** Spearman correlation coefficients between the peak posterior parameter estimates at the 0.05 level, corrected for 6*5/2 comparisons, for the baseline sample (N = 817). Correlation values below the diagonal, corrected p-values above.

	motiv.exch.rate	appet.lrn	aver.lrn	pav.bias	irr.noise	Go.bias
motiv.exch.rate	-	ns	ns	ns	***p =* 0.015**	***p =* 0.0081**
appet.lrn		-	ns	***p*< 1e-10**	ns	ns
aver.lrn			-	***p =* 8.3e-5**	ns	ns
pav.bias		***r =* -0.29**	***r =* -0.16**	-	ns	ns
irr.noise	***r = -*0.11**				-	ns
Go.bias	***r =* 0.12**					-

**Fig 6:**
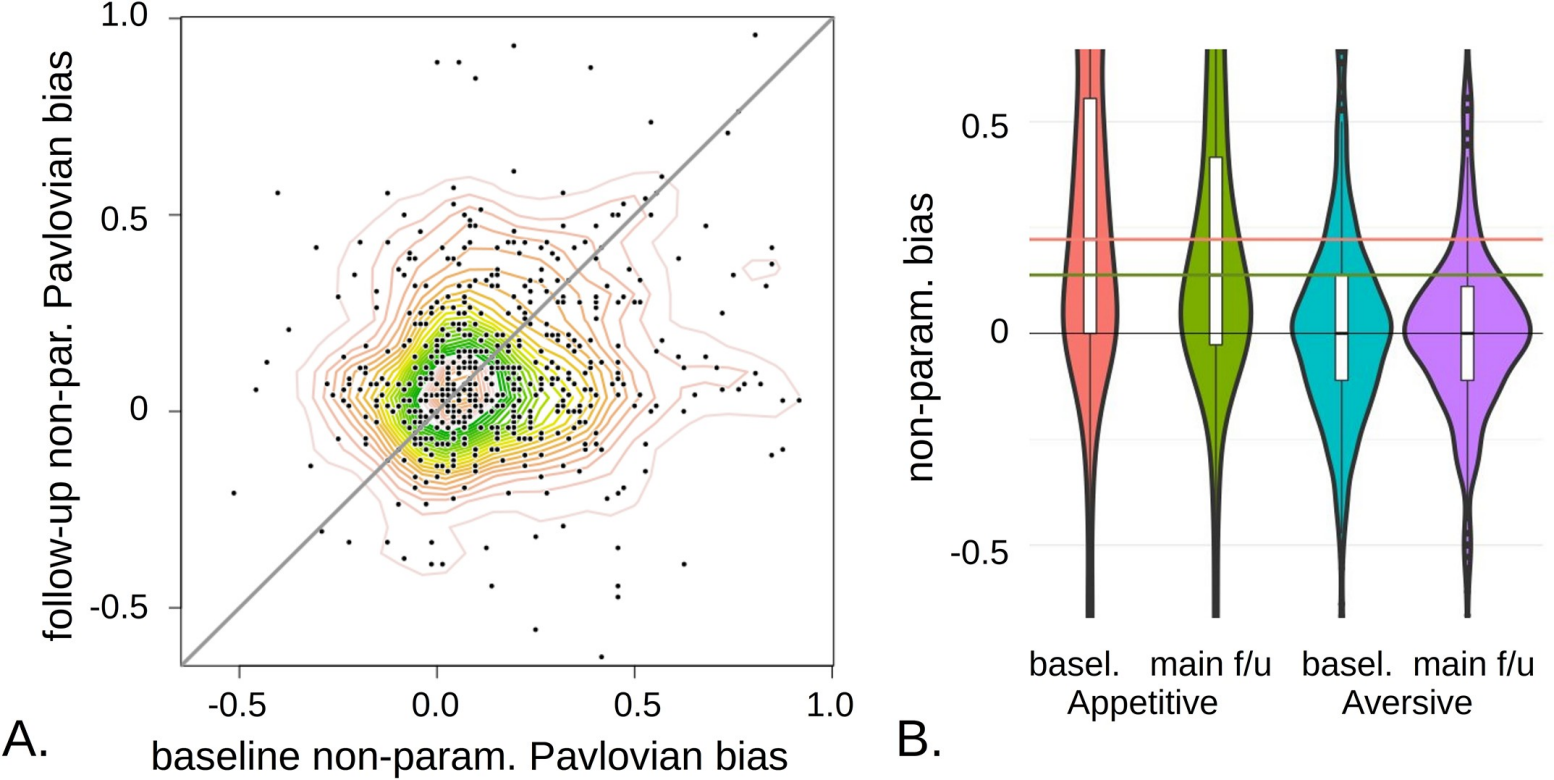
Stability in descriptive estimates of Pavlovian bias, assessed by the interaction between the fractions of total correct answers in the four conditions, ((G2W-NG2W)+(NG2AL-G2AL))/2. **A.** Stability assessed by baseline vs. long follow-up estimates. A positive correlation is detectable, rho~0.15, p = 5.4e-4. **B.** Difference in performance between the appetitive (two left) and aversive (two right) conditions. The horizontal lines show the median appetitive bias at baseline (G2W-NG2W; first violin plot; salmon) vs. long follow-up (second plot, in green). This significant difference (p = 0.0027) drives an overall reduction in the estimate of Pavlovian bias (p = 0.0019). The aversive context, NG2AL-G2AL, shows no significant change on its own (blue and mauve; p = 0.35). White boxes are interquartile ranges.

We then examined stability in the quality and nature of the performance, which is the main focus of the study. We started with descriptive measures. As suggested in Fig 3, while there was no overall change in G2W performance between baseline and follow-up (uncorr. Wilcoxon p = 0.31) the other three conditions did improve (G2AL by a median of 5.5%, p< 1e-5 corrected for 4 comparisons; NG2W by 2.8%, p = 5.4e-4; No-Go to avoid loss (NG2AL) by 2.8%, p = 1.78e-4). Next, we used these changes to compare an estimate of Pavlovian bias in follow-up vs. baseline. This estimate showed modest stability across time (Spearman *ρ* = 0.146, p = 5.4e-4; Fig 6A) but there was a significant reduction in its mean (p = 0.0019), largely attributable to a closing of the gap between G2W-NG2W (Fig 6B). Cross-sectionally, at baseline the first three conditions showed no significant linear or quadratic dependence on age (uncorr. regression p: G2W, 0.22; G2AL, 0.16; NG2W, 0.65). For NG2AL, the linear regression explained 1.0% of the variance, p = 0.021 corrected for 8 comparisons, with positive linear dependency on age. At long-follow-up, again the first three conditions showed no significant dependency at the uncorrected level (regression p: 0.98, 0.096, 0.73). However, at this time there was trend evidence for a positive linear and negative quadratic dependence of NG2AL performance with age (regression p = 0.093 corr. for 8 comparisons, adj. *r*^*2*^ = 0.012; S2 Fig).

To explore the evolution of the component cognitive processes, we examined the stability of the parameter values extracted from the fits of the winning computational model. To put the results that follow in perspective, the Pearson correlation for IQ between sessions was 0.77, p_*cor*_ << 1e-05 while for the mood measure it was 0.61, p_*cor*_ << 1e-05. A modest correlation across time points was detected for the Pavlovian bias (*r* = 0.10, p = 0.017; Fig 6A). A more salient result was an overall reduction in Pavlovian influences with session (from a median of 0.205 at baseline to 0.142 at long follow-up, p_*cor*_ *<* 1e-5). We found evidence that the short-follow-up participants changed their Pavlovian bias as much in the 6-month interval as the rest did in the mean-18-month interval. The scaled-information Bayes factor in favour of no difference was 4.01 (JZS scaled Bayes factor = 7.15, t = 0.76, p = 0.45). Decreases in Pavlovian bias between baseline and long follow-up were strongly correlated with improvements in performance in the Pavlovian-incongruent conditions, and anticorrelated with improvements in the Pavlovian-congruent conditions (S3 Table).

We then performed a set of latent change score (LCS;S3 Appendix) analyses, useful for describing longitudinal change [36]. The most complex multivariate-normal model that can be fitted to the data, known as the ‘just-identifiable model’, showed a significant dependence of change on baseline (regr. beta = -0.878, p<1e-3), with higher-bias individuals at baseline reducing their bias more at long follow up (visible in Fig 5A). This model was superior to one assuming that change only represented regression-to-mean (χ^2^ = 7.63, df = 1, p = 0.0057) and also to one assuming the same mean and variance at long follow-up vs baseline (χ^2^ = 6.37, df = 2, p = 0.041). See S3 Appendix for illustration and more details.

**Fig 7:**
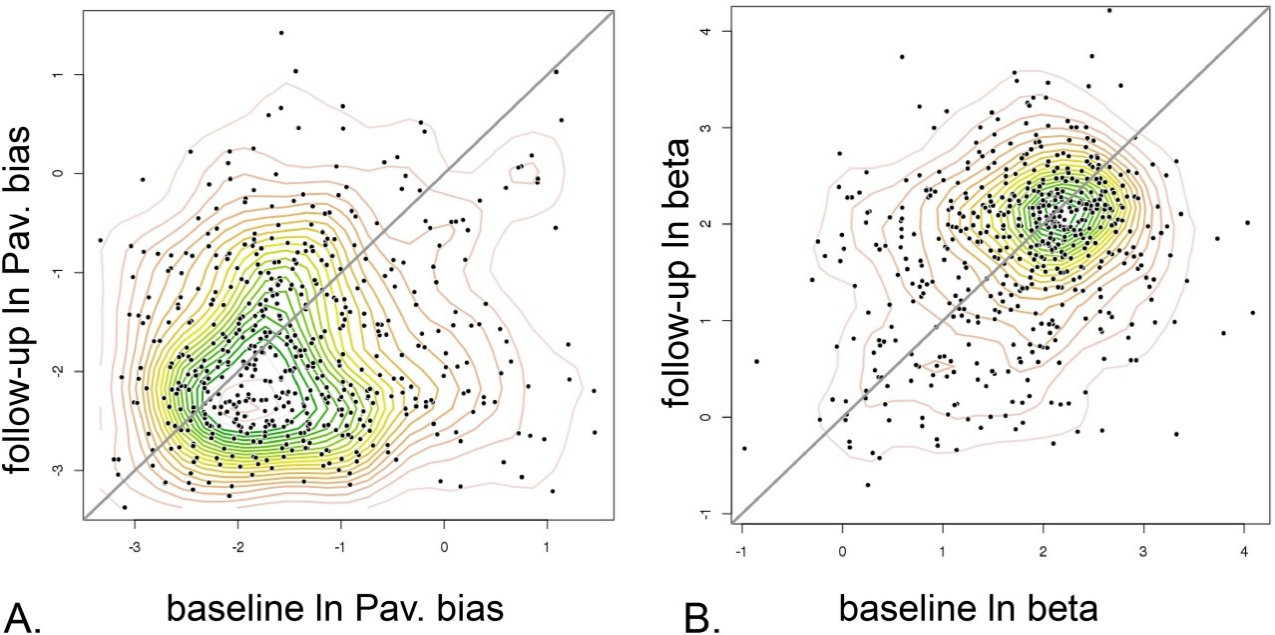
Parameter time dependency, long follow-up study, baseline vs. 18 months (mean) later. **A.** Pavlovian Bias. The most prominent feature was a reduction in the group mean. **B.** Motivational exchange rate (log beta). Here there is little shift in the modal tendency. Note that log-units are used and the contours are estimated by axis-aligned, bivariate normal kernel density estimation.

**Fig 8:**
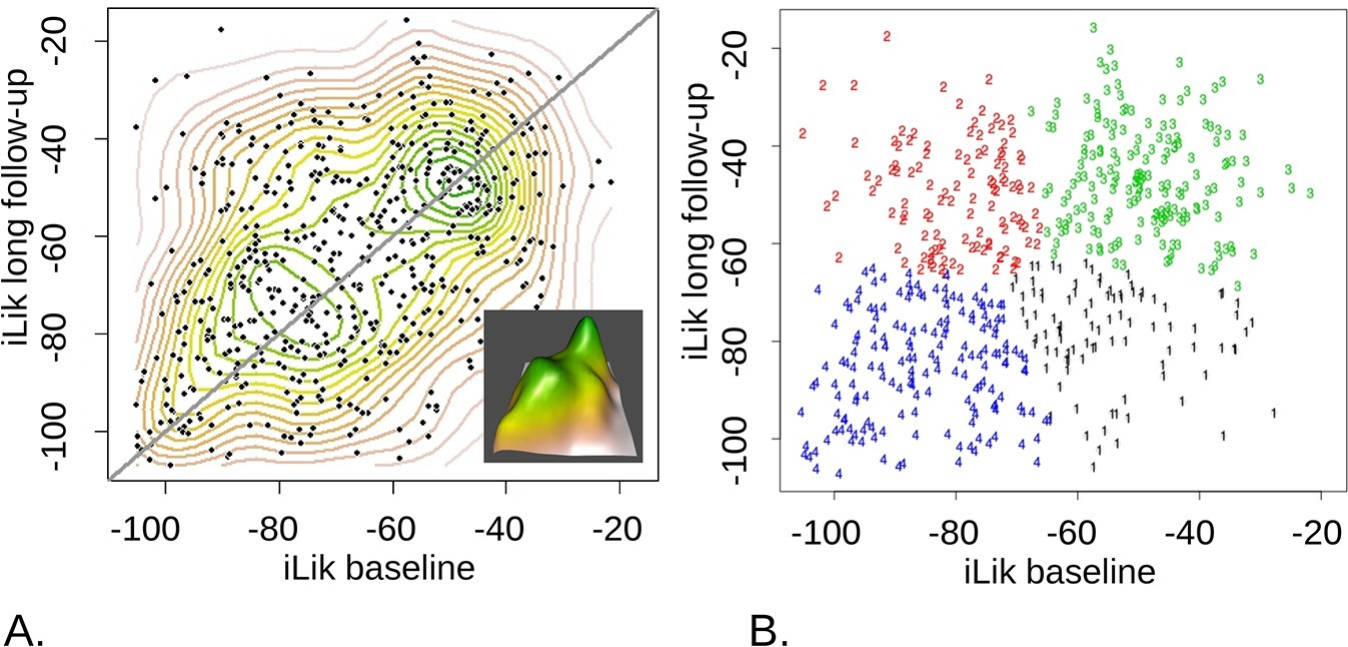
The distribution of model fit over the population is bimodal and best described by four Gaussian components. **A**. Kernel density contours are shown, while the inset plots peaks of the landscape in 3D. **B.** 4-component mixture-of-Gaussians fitted to this joint distribution of integrated likelihoods, with participants clustered according to the Gaussian they are most likely to belong to. As can also be seen in the 3D rendering. Apart from the prominent peaks (cluster3, green, N_c3_ = 176 participants, and 4, blue, N_c4_ = 171) there are somewhat less prominent concentrations (1, black, N_c1_ = 94 and 2, red, N_c2_ = 113).

We found a substantial temporal correlation for the motivational exchange rate (ln(beta); r *=* 0.253, p *= <* 1e-5; Fig 7B) and especially for the model fit measure, the integrated likelihood, (r *=* 0.37, p *<* 1e-15; Fig 8A). The latter increased at long follow-up, from a median of -68.9 to -64.4, p_*cor*_ = 0.023. There was evidence for a temporal correlation in learning rates, in both the appetitive and aversive domains (r *=* 0.09, p *=* 0.045 and r *=* 0.11, p *=* 0.011 respectively). Both increased significantly from baseline to long follow up (median differences of 0.028 and 0.027 respectively; both p_*cor*_< 1e-05). There was trend evidence of temporal correlation for the bias parameter favouring action over all trials (‘Go bias’: r *=* 0.082, p *=* 0.054) and the lapse rate parameter (r *=* 0.077, p *=* 0.068). Go-bias decreased (median 0.73 to 0.57, *p*_*cor*_ = 0.014), but the most significant change was a decrease in lapse rate (median 0.069 to 0.055, *p*_*cor*_ < 1e-05).

For none of the parameters, or their change, did we find evidence of correlation with participant age at baseline (correcting for seven comparisons). We also examined whether the baseline parameters depended on gender, mood (the ‘Mood and Feelings Questionnaire’) or IQ (WASI total IQ). Correcting for multiple comparisons, we found no significant dependency of baseline parameters on gender or mood and consequently did not analyse for such dependencies further.

IQ was significantly related to the parameters, decreasing with increasing Pavlovian bias at baseline (*r =* -0.13, *p*_*cor*_ *=* 0.007) as hypothesized. Other parameters also related significantly to IQ, notably motivational exchange rate (*r =* 0.27, *p*_*cor*_< 1e-10), appetitive learning rate (*r =* 0.11, *p*_*cor*_ = 0.044) and aversive learning rate (*r =* 0.19, *p*_*cor*_ < 1e-05). IQ strongly correlated with overall model fit (*r =* 0.28, *p*_*cor*_ *<* 1e-10). In the long follow-up sample the model fit (*r =* 0.31, *p*_*cor*_ *<* 1e-10), motivational exchange rate (*r =* 0.24, *p*_*cor*_ *<* 1e-05), and lapse rate (*r = -*0.14, *p*_*cor*_ *=* 0.014), but no other parameter, correlated with IQ. Regressing IQ on the (log) appetitive and aversive sensitivities of the valenced-sensitivity model obtained a multiple r-squared of 6.1%, whereas the single beta of the valenced-learning model absorbed 7.6% of the variance in IQ.

We then examined the distributions of parameters and of model fit for evidence of sub-grouping. We used the baseline data for this screening, as it was epidemiologically most representative. There was no evidence for sub-grouping in the parameters, but there was for the fit measures, where a bimodal distribution was evident (Fig 8A). This motivated analysis of the joint distribution of baseline and long follow-up model-fit measures. This was best described by a mixture of two major, approximately equipopulous, Gaussian components and two somewhat less prominent ones (Fig 8B). This means that the behaviour of the high-likelihood clusters is much more predictable (less random) according to our models.

We performed a mixed-effects analysis (here, controlling for re-test) to draw out differences between the clusters in terms of Mood, IQ and task parameters. As expected, we found differences between sub-groups in integrated likelihood (by construction) and motivational exchange rates and irreducible noise, which are closely related. More interesting, we found a consistent pattern across parameters and IQ, where the cluster which fit worse at both time points (blue or 4 in Fig 8) did significantly worse than most in all performance-sensitive measures, and had increased Pavlovian bias compared to clusters 2 and 3 (2 and 3 had the better fit at follow-up, Fig 8B). Pavlovian bias did not differ amongst the other clusters, while cluster 4 had greater IQ than the others. See ‘S2 Appendix: Clustering analyses’, for statistical details and illustration.

Finally, we performed *in silico* analyses to determine whether the fitting procedure could reliably recover known parameters, and whether it might introduce spurious correlations between them (see S1 Appendix, ‘In silico simulated agents’ reliability and biases’ for details). Recoverability in silico was much better than stability in vivo, suggesting that the former is not a limiting factor in our study. Similarly, no spurious correlation was observed between the Pavlovian bias and learning rates, which means that the associations between them seen in the real data are likely to reflect a true feature of the study population rather than a modelling artefact.

## Discussion

We report the first longitudinal assessment of the psychometric properties of a key computational parameter, namely the Pavlovian bias. We did this in a large, epidemiologically-based study of young people. The Go-NoGo task that we used yielded informative results in terms of the cognitive process likely to operate, the evolution over time of the parameters of that process, the construct validity of the model parameters in question and also useful methodological considerations.

We found behaviour was well fit by two models, the winning one having two different learning rates for reward and loss but a single sensitivity to returns, and the other having a single learning rate, but two different sensitivities. This is consistent with, but also finesses, recent results stressing the dependence of learning rates on outcome valence, especially in young people [37,38]. We compared models not only on the basis of approximations to the statistical evidence for each, but also on their ability to predict left-out decisions. The dual-learning model fit better, but there was no evidence for a clear allocation of model type to individuals. Model-fit improved with practice and was greater for subjects with higher IQ. Estimates of the Pavlovian bias were robust to model type, but their test-retest stability was weak, limiting inferences about individual development. At the group level, Pavlovian bias decreased when participants were re-tested 1–2 years post-baseline.

Pavlovian bias changed over test sessions, but not over age, in a characteristic pattern. In terms of hypotheses we set out to test, we interpret this as strong evidence against this parameter, as measured by this task, being a fixed stable trait. Second, we interpret a longer follow-up resulting in the same reduction in Pavlovian bias as the short one as modest evidence against this bias being a disposition slowly changing with development. The pattern is most strongly supportive of a hypothesis that Pavlovian bias approximates an experience-dependent prior expectation. Of course, our models do not directly compute the ‘probability that in an appetitive context, the correct decision is to act’, which would formally be a belief. However, an agent using such a belief would prefer Pavlovian-congruent actions, and a weakening of such a belief with experience would lead to weakening of this preference, so the evolution of Pavlovian parameters approximates beliefs or expectancies about contingency. The fact that model-fit was good and improved with time, yet parameters changed, argues against a fixed disposition. The virtual absence of cross-sectional age dependency of the parameters, and shifts being as pronounced after 6 months as after 1–2 years, argues *against* spontaneous slow development and *for* an effect of practice. Lack of strong age dependency of performance has been observed in other reinforcement learning tasks (e.g. [37] in adolescents vs. adults), but both performance and cognitive parameter age dependencies very much depend on the specific details of the task at hand [39,40]. Practice effects may have affected the improvement in the extent to which people were described by our models. Selective attrition may also have affected our data, though a follow-up rate of 68% is reassuring here. However, attrition may have rendered the long follow-up sample epidemiologically less representative than the baseline one.

In this task, the Pavlovian bias aids performance in two conditions, and harms it in the other two. If one interprets the bias as a heuristic that is generally useful, the fact that it reduces with repeated testing is consistent with its approximating a prior belief which can be at least partially overwhelmed by evidence, rather than being rigidly hard-wired by genetics and the early-childhood environment. This may apply more generally to other cognitive biases, and may happen over multiple timescales. Our study only employed a limited number of follow-up occasions, making it difficult to discern longitudinal changes *not* due to practice effects. In this study, any age, gender or mood dependency of Pavlovian bias, if present, was too subtle to resolve.

Psychometrically, the orthogonalized Go-NoGo task had the capacity to provide reliable estimates of Pavlovian bias, in that parameter recovery in silico was greater than 80%. At the same time, in human participants stability was reduced down to ~ 10–15%. The return sensitivity parameter was more stable. However, the stability of task measures was considerably lower than those for IQ and even mood, and lower than that conventionally required for tracing developmental trajectories [41].

Model fit emerged as important, over and above individual parameters, being the most stable task measure. It classified participants into clusters with discernible longitudinal trajectories (Fig 8 and S2 Appendix). The most striking differences were observed between a cluster consisting of people who showed poor at both time points (4 in Fig 8B and S2 Appendix) and the rest. These participants were characterized by a higher Pavlovian bias and lower IQ than the rest, as well as by higher decision-noise parameters. Mood did not differ between any clusters. Poorly fitting participants may have followed a strategy less well captured by our models, or may have been irreducibly more stochastic. In either case, model-fitting causes decision-variability parameters to absorb this high variance, whether its cause is hidden cognitive variables that are as yet not represented in the models, or random noise. It would be interesting to use less constrained machine-learning models to estimate the upper limit on the amount of variance in the data that cognitive-mechanism models like ours hope to explain, and thus help interpret decision variability parameters [42]. Overall, model-fit emerged as a potentially important measure to classify developmental trajectories in future research.

We found that IQ correlated with motivational exchange rates, and indeed model fit. That is, the behaviour of those with higher IQ was more affected by a unit improvement in objective outcome, over and above differences in learning and bias. This is what one would expect if IQ test scores were themselves dependent on how motivating the participants considered finding the correct answers in the IQ tests. This is in turn consistent with evidence that IQ test scores can be increased by material incentives and that motivation in the absence of additional incentives predicts real-life outcomes [43]. Likewise, confidence is linked to both IQ and motivation [44]. Our motivational exchange rates may thus reflect ability-dependent confidence, important for development during youth. Tasks involving simple inference about counter-intuitive contingencies, building on our Go-NoGo task, may be useful in exploring these relations. Furthermore, the neural process underpinning reductions in Pavlovian bias would be interesting to elucidate, as it has been suggested that top-down processes actively suppress this bias in more able participants [45]. Alternatively, reductions in Pavlovian bias occurring over time or across IQ groups in young people may relate to differences in fixed, for the duration of the experiment, Pavlovian parameters integral to associative-learning systems (*b*_*pav*_ in Eq 3) like those of our models.

Though not affecting our central hypotheses, model-comparison analyses raised important questions as to how young people learn differentially from loss and gain events. The fact that behaviour could be explained almost equally well either by recourse to valenced-learning or to valenced-sensitivity, merits further study. Learning rates and sensitivities can be distinguished [46], but the present task was not optimized to do so. A larger number of trials, for example, could help resolve this ambiguity. In terms of our models, subjects behaved either as if volatility were higher for loss compared to gain contexts (faster aversive learning), or were loss averse (higher aversive sensitivity). If appetitive and aversive sensitivities are valid concepts, they should be similar in comparable but learning-free settings, such as well-learnt versions of this task (improved from [26]). Based on the present results, we hypothesize that in a task capable of simultaneously resolving the valence-dependence of preference (valenced sensitivity) and of learning (valenced learning), the two sensitivities would be closer to each other than suggested by a pure valenced-sensitivity model, but would not be identical, as they would also be informed by loss aversion.

We studied the orthogonalized Go-NoGo task because it is well established, widely used, specialized to assess Pavlovian bias, and could provide insights (especially about decision noise and model fit) likely to be relevant to other computational tasks. However, the findings reported here should be used with caution in other contexts. Further insights about Pavlovian bias may be provided by existing paradigms [47], but testing for the putative experience-independent core of this bias with a brief task, suitable for developmental research, could be facilitated by an adaptive design. We suggest maximizing the number of plateau-performance trials by adaptively looking for the true indifference point between the effective values of the actions (*q*, not *Q*, in Eq 3). An experience-independent Pavlovian bias would mean that in the appetitive domain, the reward for the ‘No-Go’ action has to be (adaptively) boosted by an amount proportional to the mean reward of ‘Go’ and ‘No-Go’ in order to achieve indifference between these two choices. Similarly, in an aversive context the value of ‘No-Go’ has to be adaptively penalized by an amount proportional to the average of the loss returns for the two choices to achieve indifference. For symmetric average returns, the mean of the (absolute) adaptive boost and adaptive penalty components would be proportional to the Pavlovian bias, and the difference between these adaptive amounts would be the ‘Go bias’.

Methodologically, the present work extends the use of left-out-likelihood based model comparisons. These furnish outcomes that are directly intuitive and convenient for further statistical comparisons, as well as being free of approximations inherent in the BIC. On the other hand, they are much more computationally intensive and will require further refinement to render their use routine.

In conclusion, we show that Pavlovian influences characterizing young people are well described at the epidemiological level by established reinforcement-learning models. Practice and higher IQ correlated with weaker Pavlovian influences, while higher IQ scores were also associated with higher motivation to attain a given reward, suggesting important neurodevelopmental relationships. However, neither the Pavlovian bias parameter nor other key task measures met conventional psychometric standards for temporal stability or for external validity with respect to age and psychiatric symptoms, attributes useful in characterizing individual variability and individual change. It is a matter for further research whether similar problems affect other computational tasks, but our study does give urgency to the work of establishing the psychometric properties of such tasks and the parameters associated with computational models them. Studies aiming to characterize individual trajectories of decision-making will benefit from psychometrically improved computational tasks, which better exclude experience-dependent components, as well as designs that include more follow-up points.

## Methods

### The Go-NoGo task

We used an orthogonalized Go-NoGo task that contrasts a propensity to act, rather than not to do so, in context involving opportunity (‘win’) versus threat (‘avoid loss’). Participants were presented with four different abstract stimuli each of which had a constant, but unknown, association with a correct policy. The correct policy was either to emit (‘Go’) or to withhold (‘NoGo’) an action, here involving a button press. If the correct decision was made, the better of two outcomes was realised with probability 0.8. This better outcome was null (as opposed to a loss) for two stimuli and positive (as opposed to null) for the other two. The task closely followed a previously published paradigm [25], with some slight simplifications, unrelated to the core biases assessed by the task. These simplifications helped deliver it to a community sample, on a large scale and in the context of a multi-task battery. First, implementing the decision ‘Go’ was simpler, i.e. not dependent on any target features, unlike the original task in which the ‘Go’ action could be either ‘left’ or ‘right’ depending on the location of a target. This allowed trials to be shorter. However, time pressure from the remaining task battery (to be reported separately), meant that subjects performed a more restricted sample of 144 trials. Second, task clarity was improved by informing participants before performing the task that the outcome probabilities were 0.8 and 0.2. Third, motivation was made explicit by telling participants that they were playing for real money, that random performance would attract zero extra fee and excellent performance could be worth about five pounds sterling additional earnings. These changes were supported by piloting the whole battery in which the task was embedded, as we describe next.

We took precautions to ensure that the fact that the task was delivered as part of a battery did not affect the power for testing the hypotheses in question. The battery of which this task was part of consisted of 7 tasks and took over 2.5 hours to complete, whereas the task analysed here took about 23 minutes to complete, longer than the average in the battery. We first examined data from previous, longer versions of the task and performed a pilot of 15 participants. In addition to quantitative data, these participants were de-briefed in detail by trained research assistants (RAs) who interviewed them as to whether they found the tasks tiring, interesting or difficult. Although quantitative data were in line with the literature, qualitative data suggested that some participants might, subjectively, be affected by tiredness but most importantly some found the task hard to work out and felt discouraged by this. Therefore, first, the randomization of the order of tasks in the battery was constrained, so that this task took place within the first hour of testing. Second, research assistants were assertive in enforcing short breaks between tasks and emphasizing the importance of attending to the task. Third, they reminded participants that they were playing for real money and that all decisions counted approximately equally, in monetary terms, encouraging attention to each decision rather than assume that shorter tasks paid as much as longer ones. Fourth, participants were reassured that they should not be discouraged if the best answers were not clear to them as the task progressed, but on the contrary they should proceed by trial and error and the best answers were then likely to gradually ‘sink in’. This is consistent with the Rescorla-Wagner model that we used to analyse the data. After the first 50 participants were tested under careful RA supervision, an interim analysis of the whole battery and more limited feedback from RAs was reviewed. This gave no cause for concern with respect to the present task and the quantitative parameters extracted were reassuringly compatible with those from historical laboratory samples (as well as the Results reported here).

Participants were thoroughly informed about the task, including a veridical performance-related pay component.

### Participants

Community dwelling participants were recruited from within the volunteer database of the Neuroscience in Psychiatry Network Study [28].

**1. Large naturalistic study.** This consisted of N1 = 817 participants who provided task data of adequate quality for analysis. They were spread evenly across gender-balanced, two-year age bins. Age bins were used to ensure evenly spread recruitment by age, but not for analyses, where age was treated as a continuous variable. All participants formed the ‘baseline’ sample. Of these, 33 were treated for major depressive disorder (‘depression cohort’) whereas 784 were not in contact with primary or secondary mental health services, and were invited to re-attend on average 18 months later (realized interval mean = 17.79 months, SD = 3.55 months; ‘non-clinical cohort’). This non-clinical cohort was approximately representative of the local population in this age range as it was (i) balanced for sex and evenly distributed through the age range, by construction (ii) avoided high concentrations of students and used, instead, a range of representative community settings for recruitment. The follow-up rate was 71%, N2 = 557 of which 542 provided good quality data.
**2. Short follow-up study.** We tested 61 participants 6 months after baseline. These were also invited to participate in the ‘long follow-up’ (of 18 months), and 54 of them did so. There were equal numbers of participants in 5 x 2-year age bins, 14–16 years, 16–18 years etc. Each bin was balanced with respect to sex.

Volunteers were invited to be approximately equally distributed by gender and age, between the ages of 14 and 24 years old, from Cambridgeshire (60% of sample) and North London (40%). We excluded those with moderate or severe learning disability or serious neurological illness. Recruitment continued until a total sample of 820 young people agreed to participate at baseline. The Cambridge Central Research Ethics Committee approved the study (12/EE/0250). Participants gave informed consent themselves if they were at least 16 years of age, otherwise the participant was fully informed and agreed to the study, but their parent or legal guardian provided formal informed consent.

### Modelling

We explored variants of the core (here called valenced-sensitivity) model that [25] used to describe behaviour. First, the values of actions (‘*Q* values’) were calculated based on a learning rate *λ*_*v*_. We use ‘*v*’ subscripts to indicate that, in different variants of the model, the parameter in question may be valenced, i.e. different for ‘win’ and ‘avoid-loss’ trials. In the valenced-sensitivity model all trial types shared the same learning rate but different motivational exchange rates *ρ*_*v*_ were used depending on trial valence:

$$Q_{t+1}=Q_{t}(a_{t},s_{t})+\lambda_{v}(\rho_{v}r_{t}-Q_{t}(a_{t},s_{t}))$$ (1)

Only *Q* values pertaining to realized stimuli and actions were updated, with all others being carried forward from the previous trial. The model also kept track of the state values pertaining to each stimulus using the same parameters:

$$V_{t+1}=V_{t}(s_{t})+\lambda_{v}(\rho_{v}r_{t}-V_{t}(s_{t}))$$ (2)

Crucially, the conventional *Q* values were biased by two terms representing an overall tendency towards action (‘Go bias’) and a Pavlovian bias (towards action or inaction) that depended on valence (state value):

$$q_{t}(a_{t},s_{t})=Q_{t}(a_{t},s_{t})+b_{go}(a_{t})+b_{pav}(a_{t})V_{t}(s_{t})$$ (3)

where the two bias coefficients *b* are zero unless $a_{t}=Go$. In effect this means that the No-Go action was taken as a comparator, and the Go action was either penalized (in aversive contexts) or boosted (in appetitive ones) proportional to the value of the respective stimulus. Finally, the policy probability for choosing an action was given by the softmax function, modulated by a lapse rate parameter *ξ*:

$$p(a_{t}|s_{t})=(1-\xi )\frac{\exp(q(a_{t},s_{t}))}{\sum_{k}(exp(q(a_{k},s_{t}))}+\frac{\xi }{2}$$ (4)

Thus the motivational exchange rates *ρ*_*v*_ acted as an inverse temperature parameter for the softmax by scaling the outcome values in Eq 1, which then fed into Eq 4 via Eq 3.

Several model variants were explored. First, separate learning rates were used depending on valence (‘valenced-learning model’) with a single outcome sensitivity.

Second, an additional memory/forgetting parameter was introduced such that *Q* values pertaining to unexperienced state-action pairs decayed by a constant fraction per trial, rather than being carried forward intact (‘forgetting model’), quantified by an additional ‘memory’ or ‘forgetting’ parameter [48]. In these ‘forgetting models’ models, we argued that during long periods when, by chance, a particular stimulus was not observed, the associated actions might drift back to zero if participants brought to the task a (strictly unjustified, but common-sensical) assumption that values might be subject to non-zero volatility. The other models assumed that the value of stimuli not seen in a particular trial would not change.

Third, the appetitive and aversive sensitivities of the valenced-sensitivity model were transformed into an overall sensitivity and an appetitive/aversive ratio (‘sensitivity ratio model’). This had two motivational exchange parameters, just like the valenced sensitivity model, but formulated them as an appetitive sensitivity and a sensitivity ratio. Therefore, at the level of the individual it was identical to the valenced sensitivity model. At the group level, the expectation-maximization fit used prior distributions for the parameters that were independent of each other. In the case of the valenced sensitivity model, this means that the distribution of appetitive sensitivity over the population was modelled as independent to the aversive one. For the sensitivity-ratio model, a positive mean for the population distribution of the eponymous parameter would encode a positive correlation between appetitive and aversive exchange rates. Model fits favoured the former model.

### Generative model fitting

We used hierarchical type-2-ML model-fitting, assuming that each wave of data could be described by a set of independent prior distributions for the mean and spread of each parameter. Waves of testing were fitted independently. Individuals’ parameters were optimized given point estimates of the mean and spread of the group they belonged to, which were themselves re-estimated. Specifically, we used the expectation-maximization algorithm in [25], modifying MATLAB [49] code provided by Dr. Quentin Huys. We estimated the ‘integrated likelihood’ and ‘integrated BIC’ measures used in that work (Eq 5), using the same sampling technique.

$$\begin{array}{l} iL_{pt}=\text{ln}\int p(d_{pt}|\theta ,Μ)p(\theta |\Theta )d\theta \\ iBIC=-2\sum_{pt}iL_{pt}+N_{par}\text{ln}(n_{tr}n_{pt}) \end{array}$$ (5)

Where *iL*_*pt*_ is the integrated likelihood for participant *pt*, *d*_*pt*_ the data provided by this participant, *θ* the ‘micro’ parameters of that participant according to model M, Θ the ‘macro’ parameters describing the population distribution of *θ*, *N*_*par*_ the length of Θ, *n*_*tr*_ the length of *d* (same for each participant) and *n*_*pt*_ the number of participants.

For the purposes of model-fitting each parameter was transformed according to theoretical assumptions and the group distribution approximated by a Gaussian in the transformed space. For example, following [25] we assumed that $b_{pav}>0$ so this parameter was fitted in log space, whereas parameters having both an upper and lower bound were logistic-transformed. We tested whether the model-fitting procedure was robust to assumptions about the distribution of parameter values in the population (S1 Appendix). We tested two assumptions that were particularly relevant to Pavlovian bias. First, we allowed the population distribution of Pavlovian bias to be normal rather than log-normal. Using mean and variance population estimates derived from the real data, this translated to the presence of a small tail of negative values (S1 Appendix). Second, we tested whether model-fitting was sensitive to the precise form of the population distributions used, e.g. gamma rather than log-normal. Recovery of Pavlovian bias parameters was reassuringly robust in the face of such twists (S1 Appendix).

### Out-of-sample testing

In order to go beyond comparing models by using simple approximations like the BIC, we argued that better models should provide a higher likelihood for data on which they were not trained, compared to less good models. To do this, we fitted models as best as possible to everyone, leaving out certain test trials. Then we compared the sum-log-probability of the actual responses participants provided on these left-out trials, thus performing paired left-out-likelihood (LOL) comparisons. Confidence intervals around this difference of predictability-per-trial provide an intuitive measure of how much better one model is than another, especially when compared to the average predictability-per-participant-decision.

For the LOL comparison to be optimal, models must be given the best possible chance to describe the individuals whose parameters are used to derive the LOLs. In order to do this, we first divided the sample into a 300-strong ‘group training’ set and a test set. The ‘group training set’ was used to provide the best possible descriptive statistics of the entire population in terms of the means and variances of each (transformed) parameter. These were the group-level parameters fitted by the type-2 maximum-likelihood procedure (S3 Fig).

We then used these group-level parameters to provide priors for fitting the remaining ‘test set’, from which the ‘test trials’ were left out. Markov-chain monte-carlo (MCMC) fitting at the level of the individual participant was performed, using the sum-log-posterior over the included, non- left-out, trials only to derive posterior beliefs about parameters for each participant in the test sample. During fitting, MCMC efficiently provided sum-log-likelihood samples over the left-out-trials, thus forming the integrated LOL. Two trials were left-out, as described below. LOLs were not taken into account for parameter estimation, to avoid double-dipping. We repeated the procedure with different candidate models, thus obtaining (paired) model comparisons of their predictive power over the hidden trials only.

For optimal performance, we first decided how many trials to leave out (left-out trials, LOTs). When a limited amount of data per participant is available, greater numbers of LOTs result in noisier parameter estimates for each model, making it more difficult to detect differences between models. Furthermore, it is not a priori known how model fit may deteriorate as a function of the number of LOTs for different models. Hence, it makes sense to use the minimum number of LOTs and rely on our high number of participants to power model comparisons. In order to assess whether these considerations were important in practice, we generated synthetic data using the valenced-sensitivity model, the best in the literature. Using synthetic data we compared LOL using the procedure above with the true LOL according to the generative parameters. S4 Fig shows how increasing the number of LOTs significantly degraded the power of the true generative model to explain LOT data.

Next, we assessed the effect of learning on LOL estimation. Because learning occurs in every trial, learners follow different trajectories in the included trials depending on what happens in the left-out trials. Thus, even if the LOL is not used during model fitting, information from the LOTs may influence the fitted parameters, thus potentially biasing fitted parameters towards values most consistent with the participant’s choices (and hence high likelihood thereof) in the LOTs. Thankfully, we do not have to guard against every possible such influence, but only to make sure that using information from the LOTs (which stimuli where shown, which responses were performed and which returns were obtained) does not unduly bias the estimation of parameters based on the included trials towards values that make the LOTs appear more likely. In order to reassure ourselves about this, we performed a series of numerical experiments where we compared using the information above, to marginalizing over the above responses and rewards.

Consider a model M with a single parameter *ε*. Assume, furthermore, that we have a flat prior $p_{0}(\varepsilon )\sim 1$ over this parameter. If h is the left-out, or hidden, decision data and v is the included, or visible to the model, data, taking into account the flat prior gives:

$$\begin{array}{l} p(h|v,M)=\sum_{\varepsilon }p(h,\varepsilon |v,M)\\ =\sum_{\varepsilon }p(h|\varepsilon ,M)\frac{p(v|\varepsilon ,M)}{\sum_{\varepsilon '}p(v|\varepsilon ',M)} \end{array}$$ (6)

The question is how *p*(v|*ε*,*M*) ought to be calculated in order not to bias estimation of how good model *M* is, i.e. not to bias *p*(*h*|v,*M*). As learning takes place from trial to trial, should the 'gaps' in v be filled in with the veritable choices of the participant, or be marginalized over? To investigate this matter, we first performed a numerical experiment with a simple Rescorla-Wagner model with learning parameter *ε*, making binary choices between alternatives via a softmax function of known parameter τ = 0.1 (i.e. a bare-bones version of our models). We generated 10000 x 28 trial epochs, for three levels of ε = 0.05, 0.15 and 0.25. Returns were deterministic returns (action1 → 1, action2 → 5), starting values for each run: *Q*_0_(action1) = *Q*_0_(action2) = 0. The first 8 trials were hidden. We looked for bias by examining how our estimate of *p*(*h*|v,*M*) depended on whether *p*(v|ε,*M*) is estimated using ‘informed’ visible trials, *p*_(i)_(v|ε,*M*), or ‘agnostic’ ones (i.e., marginalizing over hidden trials), *p*_(a)_(v|ε,*M*). A typical example of how *p*_(i)_(v|ε,*M*) may differ from *p*_(a)_(v|ε,*M*) is shown in S5 Fig. Although they gave different results for each individual subject, there was no difference (and no bias) with respect to the estimates over the hidden trials for any level of ε examined. This is shown in S6A Fig. It is interesting to note that some simple measures were biased in the expected way; for example, the maximum-likelihood estimate of the learning rate based on the ‘informed’ method was closer to the maximum-likelihood estimate over the hidden trials compared to the equivalent ‘agnostic’ estimate. In the case of ε = 0.15 this was by 0.03 log units, Wilcoxon p < 1e-08.

We then examined two further models, an *η*-greedy learner and an ‘observation-violating *η*-greedy’ learner. The latter was similar to the former, but, importantly, only updated action values for exploratory actions if they furnished a better-than-expected prediction error. We did not detect any bias in the simple *η*-greedy but we found a very small bias in the expected direction for the observation-violating model. The bias corresponded to 0.45% of the grand mean prediction probability. Given that this sequence of models was designed to showcase a difference between the more rigorous agnostic and the more practical informed approach, we concluded that any bias introduced by using the informed approach on our real data would be negligible.

### Longitudinal change models

We first compared longitudinal change with paired nonparametric tests. We also examined change using latent-change-score (LCS) models [36]. To do this we transformed the distributions of each (already transformed as above) parameter at each timepoint to normal as described below. LCS models formulate the change between baseline and follow-up as a latent variable, and estimate its mean, variance but also its possible dependence on baseline values. Pure regression towards the mean endows this change parameter with a value of -1. We used BIC and the likelihood ratio test to compare this model with nested, simpler models of change in the population distribution of parameters. Before we applied the latent-change-score formulation (S3 Appendix), we forced the marginal distributions of the transformed parameters into a Gaussian form. This further transformation-to-Gaussian to was achieved by first, estimating the mean and SD of the parameter distribution in question. Second, estimating an empirical (stepped) cumulative distribution function for this parameter with the R function edcf [50]. Third, applying an inverse-gaussian-cdf with the same mean and SD as the original. We then applied the just-identified univariate latent change score model.

This formulation does not extract from the data more than the statistics we might otherwise estimate–i.e. the means, variances and covariance of the baseline and long follow up measures, if we assume a bivariate normal distribution. It is however convenient in order to focus on change and phrase different hypotheses in terms of model comparison (BIC, likelihood ratio etc.).

### Measures of general intelligence and symptoms

We used the ‘Mood and Feelings Questionnaire’ (MFQ) as measure of mood and the Revised Children’s Manifest Anxiety Scale—2 (RCMAS) as a measure of anxiety [28,51,52]. General intelligence was measured by the full-scale IQ of the Wechsler Abbreviated Scale of Intelligence [53]. Measurements of IQ were performed on the same day as the task for the naturalistic longitudinal study. We used MFQ measurements taken near to the baseline testing session.

## Supporting information

S1 Fig In this sample, the ‘Mood and Feelings Questionnaire’ shares over 90% of the variance of the ‘general psychopathology factor’ (St. Claire et al, 2017). The relationship is monotonic and the linear part of the relationship already captures 83% of the variance, but here the minor but still highly significant quadratic and cubic components are included in green. We are thus justified to use MFQ itself as a simple and easier to interpret proxy for general propensity to experience psychiatric symptoms.
(PDF)

S2 Fig Age dependence of performance. Here, performance in ‘No-Go to avoid Loss’ is shown. There is an overall increase in performance with age.
(PDF)

S3 Fig Schematic of the algorithm used to ensure that the out-of-sample likelihood to be used for model comparison was tightly constrained but did not itself inform estimation of parameters used for its own derivation. Some steps are ‘good enough’ rather than fully optimal (but computationally very costly). For example, the derivation of priors on the test group parameters could have used the individual-fit trials, but to very little benefit.
(PDF)

S4 Fig Quality of fit as a function of number of left-out-trials. Blue lines: 95% bootstrap confidence interval (BCI) for the likelihood of included trials according to the true, valenced-sensitivity model as the number of left-out trials increases. 300 synthetic ‘training’ participants and 100 ‘test’ participants are used. The included trial likelihood is not significantly affected within this range, whose high end corresponds to 25% of all trials. Red lines: The BCI excludes the true LOL as soon as more than 2 trials per participant are left out.
(PDF)

S5 Fig Typical example of the dependence of the estimate of the likelihood on whether the estimation is informed by the actual choices in the hidden trials or marginalized. (PDF)

S6 Fig Example distributions of the difference in predictability of the hidden trials for two ways of accounting for the decisions over hidden trials when estimating parameters. (PDF)

S1 Appendix In silico simulated agents’ reliability and biases. (PDF)

S2 Appendix Clustering analyses. (PDF)

S3 Appendix Latent change score modelling. (PDF)

S1 Data Data file with baseline Pavlovian Bias in Youth task data. This includes all the raw data on which the analyses are based, from the ‘Baseline sample’. See text for details. The key matlab scripts for these analyses, provided below, operate on the unzipped data (i.e. ‘mat’ files).
(ZIP)

S2 Data Data file with short follow-up Pavlovian bias in Youth task data. As above, but for the 62 participants that participated in the 6-month follow up.
(ZIP)

S3 Data Data file long follow-up Pavlovian bias in Youth task data. As for S2 Data, but for the large, 18-month long follow up sample.
(ZIP)

S4 Data Zip file with matlab analysis scripts’. This includes the ‘master script’ GNGmodelFit2, that can be used to fit a number of different models by expectation maximization and forms the core of the analyses in the main paper. It can be used with a number of models also provided. These are functions that provide the likelihood (ll) for different models. For example, ‘ll2baepxbm’ stands for ‘two-beta, one-learning-rate alpha, one lapse rate ‘e’, one pavlovian bias ‘p’, one go-bias (the second ‘b’), one memory parameter ‘m’. In contrast, ‘llb2a…’ is the model with two learning rates, called ‘valenced learning’ in the text. Finally, three functions are provided to generate simulated data and re-fit them. simGNG1b2a simulates valenced learning, and simGNG1bba valenced sensitivity data. The function GNGmodelFit_4sims is can be used to re-fit generated data, either with the ‘correct’, generative, or an alternative model.
(ZIP)

S1 Table Neuroscience in Psychiatry Network Study & consortium author list. (PDF)

S2 Table Posterior spearman correlations at the 0.01 level, corrected for 6*5/2 comparisons, for the baseline sample, for the valenced sensitivity model. Correlation values below the diagonal, corrected *p* values above. Note the absence of significant correlation between appetitive and aversive sensitivity, in line with the worse fitting of the sensitivity-ratio model compared to the valenced-sensitivity one.
(PDF)

S3 Table Support for the theoretically expected relationship between Pavlovian bias and performance. Other things being equal, a decrease in Pavlovian bias should improve performance in the Pavlovian-inconsistent conditions (+ve pearson r by convention in this table) and decrease performance in the Pavlovian-consistent conditions (-ve r). To test this, we tested the hypothesis on each condition separately, adopting a multiple comparison threshold of p = 0.05/4 = 0.0125. We found significant evidence for all expected correlations.
(PDF)
