# Supplementary material for: Change, stability, and instability in the Pavlovian guidance of behaviour from adolescence to young adulthood
Source: PLoS Comput Biol. 2018 Dec 31;14(12):e1006679. doi: 10.1371/journal.pcbi.1006679 (PMC6329529; doi:10.1371/journal.pcbi.1006679)
Supplement: S1 Fig — The relationship is monotonic and the linear part of the relationship already captures 83% of the variance, but here the minor but still highly significant quadratic and cubic components are included in green. We are thus justified to use MFQ itself as a simple and easier to interpret proxy for general propensity to experience psychiatric symptoms. (PDF) [file pcbi.1006679.s001.pdf]

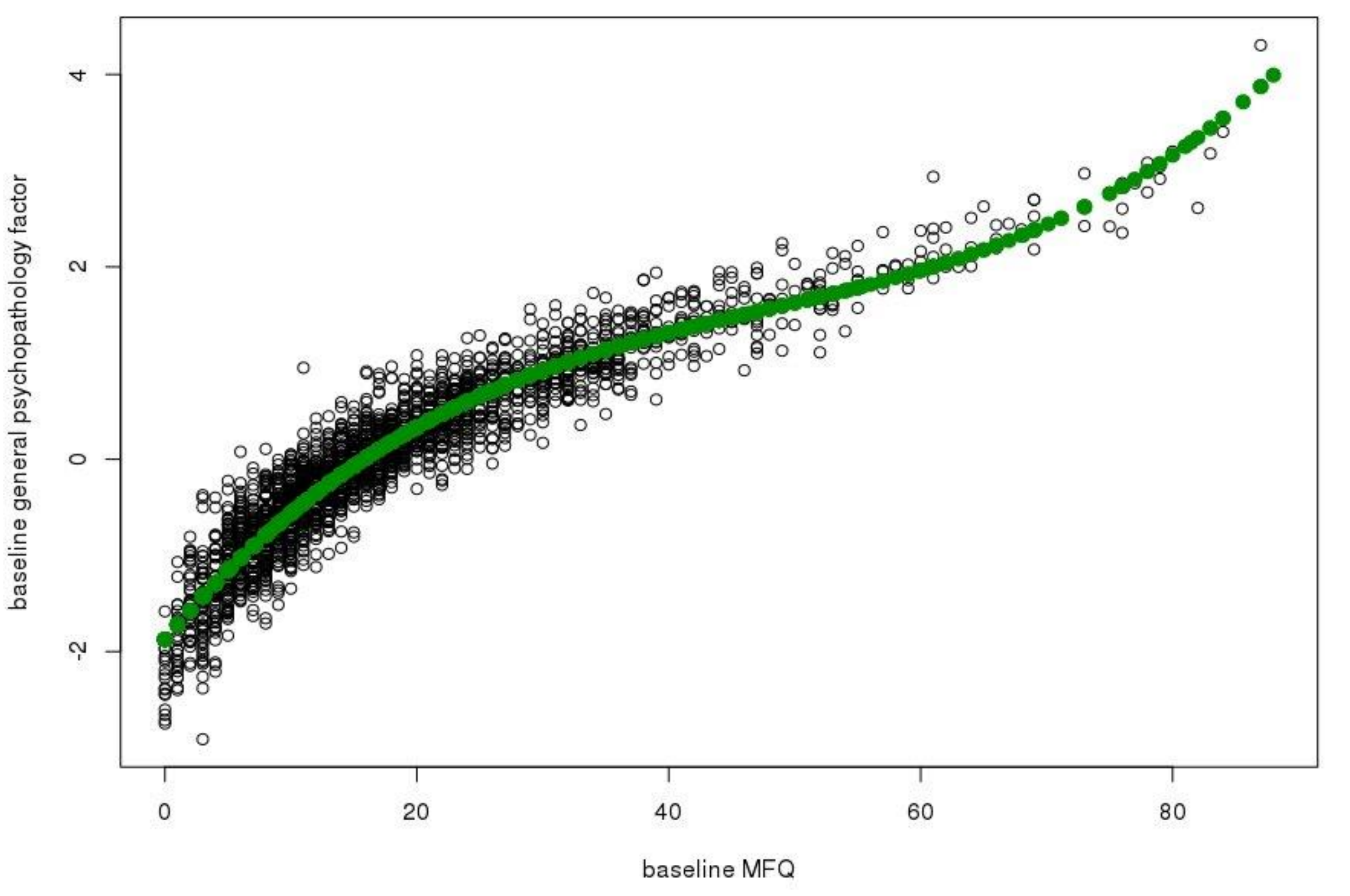


Fig S1. In this sample, the ‘Mood and Feelings Questionnaire’ shares over 90% of the variance of the ‘General psychopathology factor’ (St. Claire et al, 2017). The relationship is monotonic and the linear part of the relationship already captures 83% of the variance, but here the minor but still highly significant quadratic and cubic components are included in green. We are thus justified to use MFQ itself as a simple and easier to interpret proxy for general propensity to experience psychiatric symptoms.
