# Supplementary material for: Change, stability, and instability in the Pavlovian guidance of behaviour from adolescence to young adulthood
Source: PLoS Comput Biol. 2018 Dec 31;14(12):e1006679. doi: 10.1371/journal.pcbi.1006679 (PMC6329529; doi:10.1371/journal.pcbi.1006679)
Supplement: S2 Fig — Here, performance in ‘No-Go to avoid Loss’ is shown. There is an overall increase in performance with age. (PDF) [file pcbi.1006679.s002.pdf]

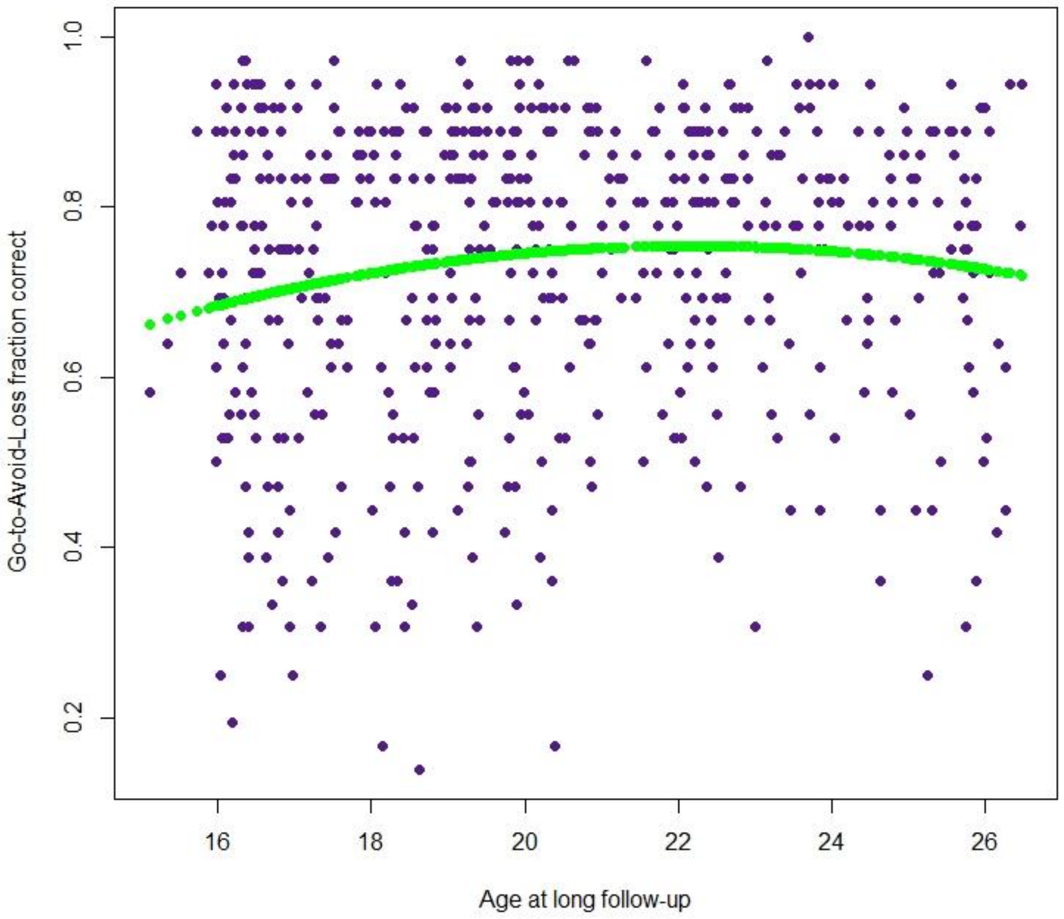

Fig S2. Age dependence of performance. Here, performance in ‘No-Go to avoid Loss’ is shown. There is an overall increase in performance with age.
