# Supplementary material for: Change, stability, and instability in the Pavlovian guidance of behaviour from adolescence to young adulthood
Source: PLoS Comput Biol. 2018 Dec 31;14(12):e1006679. doi: 10.1371/journal.pcbi.1006679 (PMC6329529; doi:10.1371/journal.pcbi.1006679)
Supplement: S3 Fig — Some steps are ‘good enough’ rather than fully optimal (but computationally very costly). For example, the derivation of priors on the test group parameters could have used the individual-fit trials, but to very little benefit. (PDF) [file pcbi.1006679.s003.pdf]

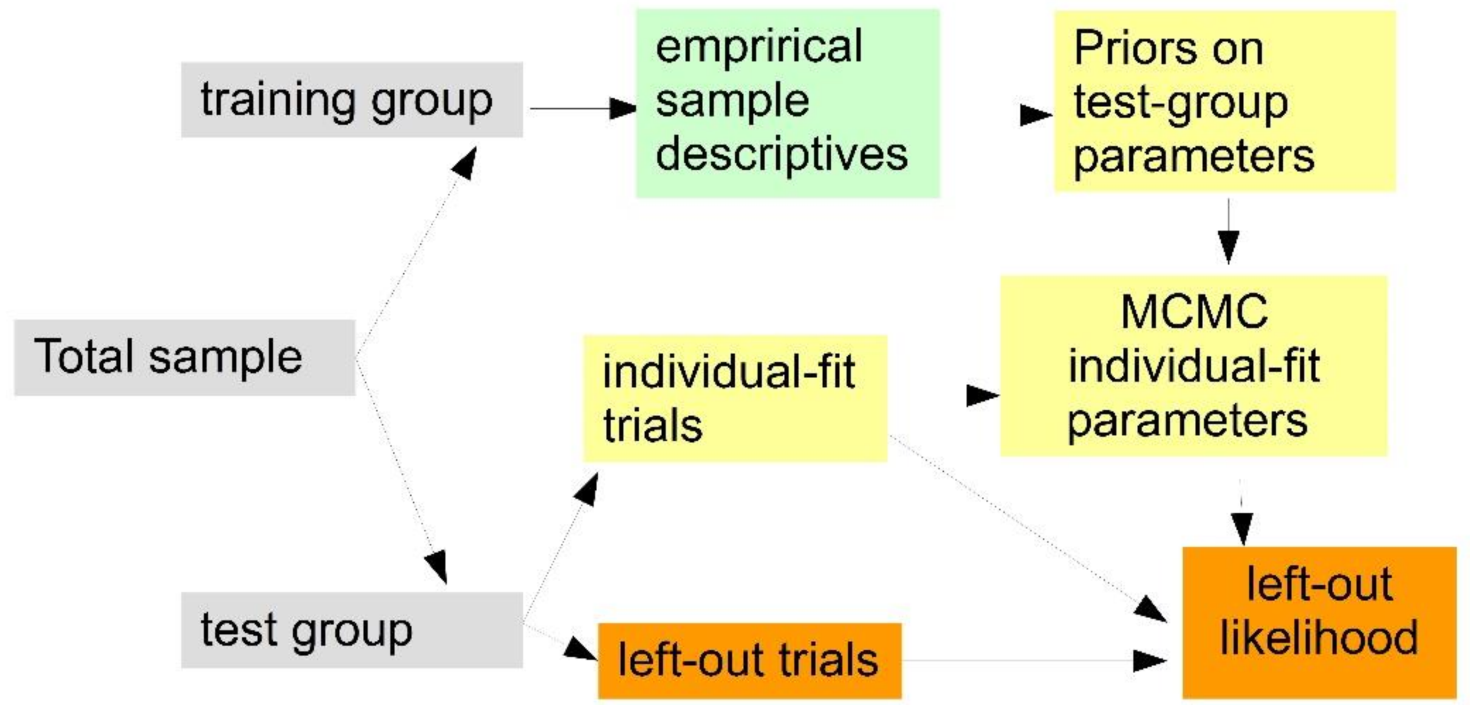

Fig S3. Schematic of the algorithm used to ensure that the out-of-sample likelihood to be used for model comparison was tightly constrained but did not itself inform estimation of parameters used for its own derivation. Some steps are ‘good enough’ rather than fully optimal (but computationally very costly). For example, the derivation of priors on the test group parameters could have used the individual-fit trials, but to very little benefit.
