# Supplementary material for: Change, stability, and instability in the Pavlovian guidance of behaviour from adolescence to young adulthood
Source: PLoS Comput Biol. 2018 Dec 31;14(12):e1006679. doi: 10.1371/journal.pcbi.1006679 (PMC6329529; doi:10.1371/journal.pcbi.1006679)
Supplement: S4 Fig — Blue lines: 95% bootstrap confidence interval (BCI) for the likelihood of included trials according to the true, valenced-sensitivity model as the number of left-out trials increases. 300 synthetic ‘training’ participants and 100 ‘test’ participants are used. The included trial likelihood is not significantly affected within this range, whose high end corresponds to 25% of all trials. Red lines: The BCI excludes the true LOL as soon as more than 2 trials per participant are left out. (PDF) [file pcbi.1006679.s004.pdf]

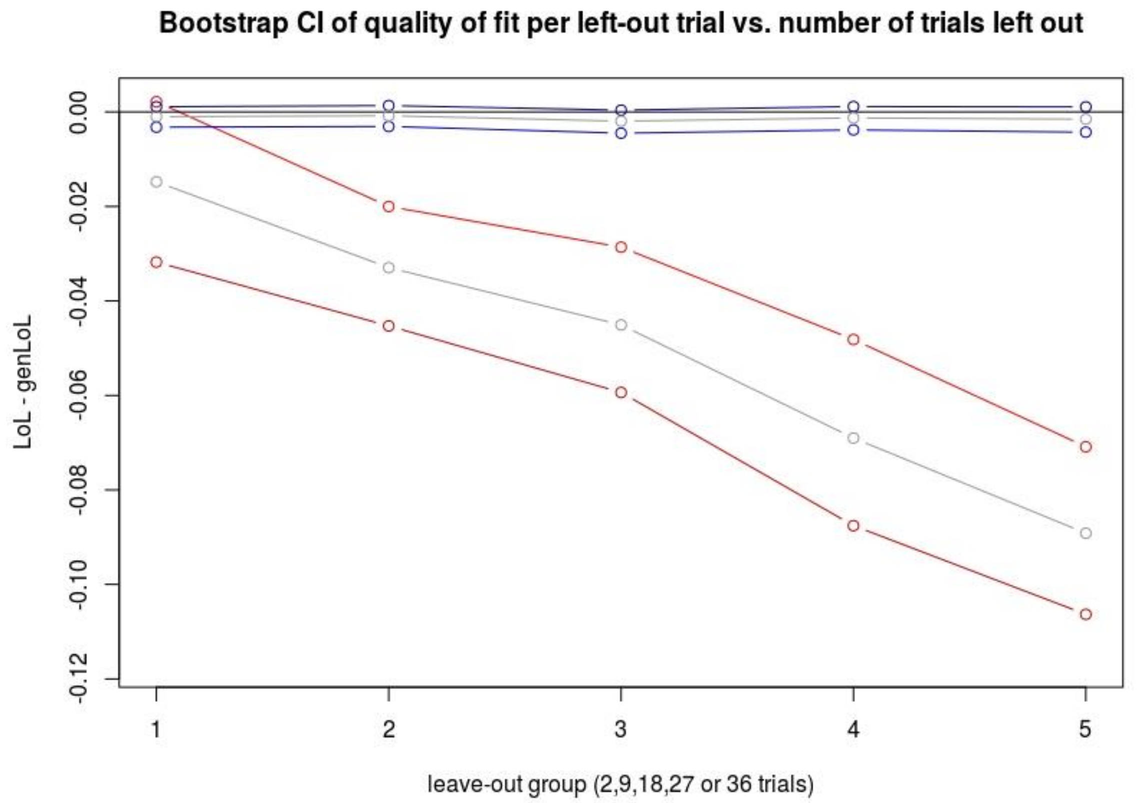

Fig S4. Blue lines: 95% bootstrap confidence interval (BCI) for the likelihood of included trials according to the true, valenced-sensitivity model as the number of left-out trials increases. 300 synthetic ‘training’ participants and 100 ‘test’ participants are used. The included trial likelihood is not significantly affected within this range, whose high end corresponds to 25% of all trials. Red lines: The BCI excludes the true LOL as soon as more than 2 trials per participant are left out.
