# Supplementary material for: Change, stability, and instability in the Pavlovian guidance of behaviour from adolescence to young adulthood
Source: PLoS Comput Biol. 2018 Dec 31;14(12):e1006679. doi: 10.1371/journal.pcbi.1006679 (PMC6329529; doi:10.1371/journal.pcbi.1006679)
Supplement: S5 Fig — (PDF) [file pcbi.1006679.s005.pdf]

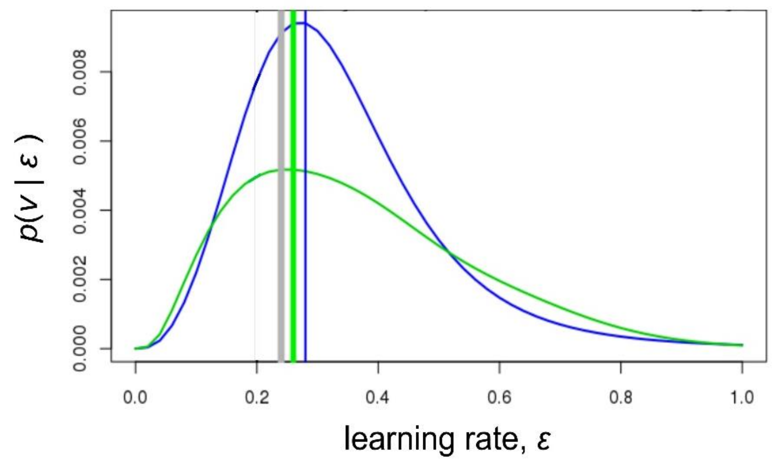

a.

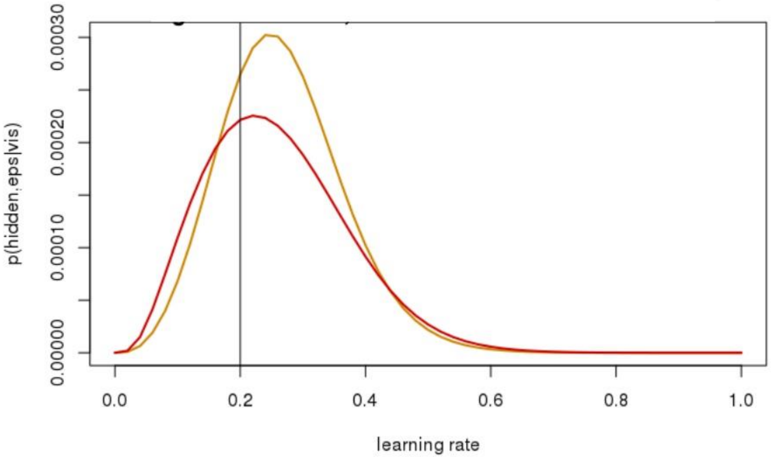

b.

Fig S5 . Typical example of the dependence of the estimate of the likelihood on whether the estimation is informed by the actual choices in the hidden trials (blue) or marginalized (green). In this example the maximum-likelihood learning rate is somewhat lower for the agnostic estimation. True learning rate here was 0.15. **a.** Likelihood over visible trials, blue = informed, green = marginalized. The vertical lines show exactly where the peaks of each distribution are. Grey is the ML value estimated over all trials, with the corresponding full distribution omitted for clarity. **b**. Likelihood over hidden trials, gold = informed, red=marginalized. The thin vertical line is again the simple ML estimate, but this time for the hidden trials ones only .
