# Supplementary material for: Change, stability, and instability in the Pavlovian guidance of behaviour from adolescence to young adulthood
Source: PLoS Comput Biol. 2018 Dec 31;14(12):e1006679. doi: 10.1371/journal.pcbi.1006679 (PMC6329529; doi:10.1371/journal.pcbi.1006679)
Supplement: S6 Fig — (PDF) [file pcbi.1006679.s006.pdf]

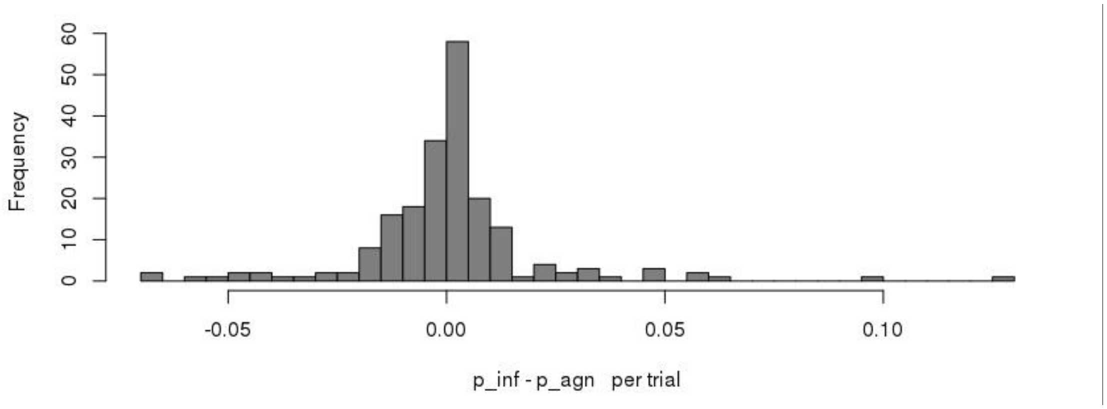

a.

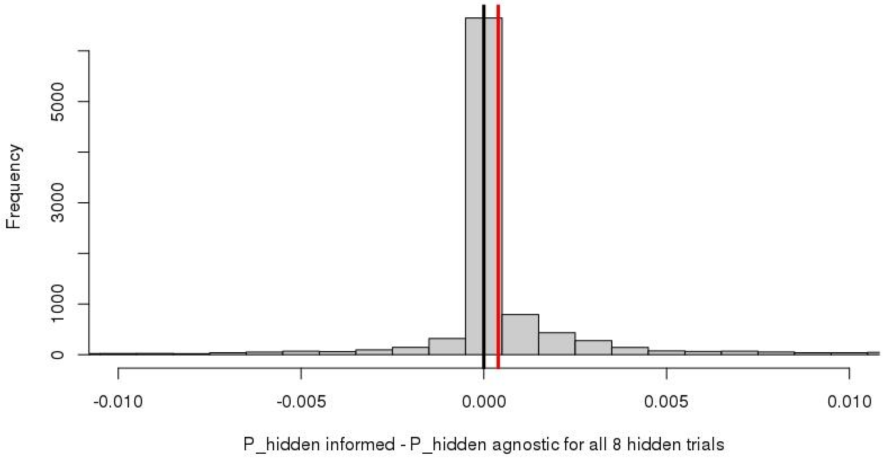

b.

Fig S6 Example distributions of the difference in predictability of the hidden trials for two ways of accounting for the decisions over hidden trials when estimating parameters. **a.** RW model, true learning rate=0.15, N=1e3 simulated participants. The geometric mean of the predictive probability per trial for the informed (Ppt_inf) vs. agnostic (marginalized, Ppt_agn) methods is shown. Not only is there no statistically significant difference (Wilcoxon $p = 0.79$), but the mean predictability achieved by the two methods is almost identical. **b.** Observation-violating η-greedy model, true learning rate = 0.24, N=1e4. Here there is a very small but statistically significant bias for the informed model to make optimistic estimates of the probability over hidden trials. Red = mean bias ~ 3.9e-4 over 8 hidden trials, $p < 1e-7$.
