# Supplementary material for: Change, stability, and instability in the Pavlovian guidance of behaviour from adolescence to young adulthood
Source: PLoS Comput Biol. 2018 Dec 31;14(12):e1006679. doi: 10.1371/journal.pcbi.1006679 (PMC6329529; doi:10.1371/journal.pcbi.1006679)
Supplement: S1 Appendix — (PDF) [file pcbi.1006679.s007.pdf]

Appendix S7. In silico simulated agents' reliability and biases

We assessed whether our model-derived parameter measures were sensitive, reliable and discriminative over the ranges of magnitude we encountered in the real data. We used individual parameters sampled from population-level distributions derived from the real data, generating N=100 or more, enough to give $p < 5e-4$ for each correlation coefficient reported here. Reassuringly, re-fitted parameters were strongly correlated with the generative ones. However, the reliability with which different parameters could be retrieved was variable. In all variants of the models considered (valenced-sensitivity, valenced-learning, forgetting), re-fitted Pavlovian bias parameters correlated with true ones with $r \sim 0.8 – 0.85$ (Fig. S8, S11-S13). Go-bias parameters were even more retrievable ($r \sim 0.9$ in the two best models). Parameters which were not as central to the phenomena that the task was developed to assess were retrieved less well, including lapse rate, ($r \sim 0.47$ and ~ 0.25 in the dual-learning without- or with-forgetting versions). On the contrary, in a sample of 300 simulated participants, no correlations between different generative vs. re-fitted parameters, for example generative action bias vs. refitted Pavlovian bias were significant under Bonferroni correction, and no pair shared more than 2.5% of variance.

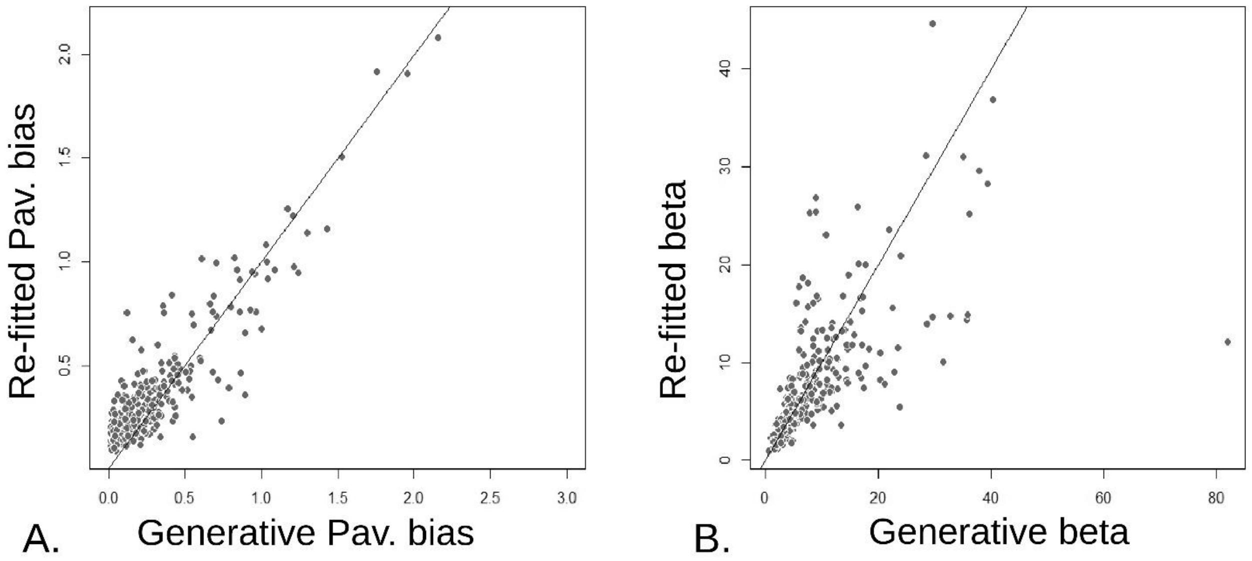

Figure S8. Simulated, then re-fitted parameters based on the valenced-learning model without forgetting, using group-level generative parameters derived from the fit to the baseline sample. The generative and fitted models utilise the same parametric form of the group level distributions. In each plot the straight line is the identity map. See figures S11 – S13 for additional simulation-refit results. **A.** Pavlovian Bias is retrieved quite well overall. r=0.80, p < 1e-05 **B.** Motivational exchange rate is retrieved somewhat less well (r=0.66, p < 1e-05 including outlier)

As with the real data, the best two models were not easy to distinguish *in silico* (Fig. S9). For baseline-like data produced by the valenced-learning model, the valenced-sensitivity model had an integrated likelihood which was 0.29 log units worse per participant (mean -65.12 vs. -65.43). This measure misclassified 35% of participants (Fig S9A). For data produced by the valenced-sensitivity model, the valenced-learning model was 0.33 log units worse per participant (mean –67.33 vs -67.67; misclassification= 47%).

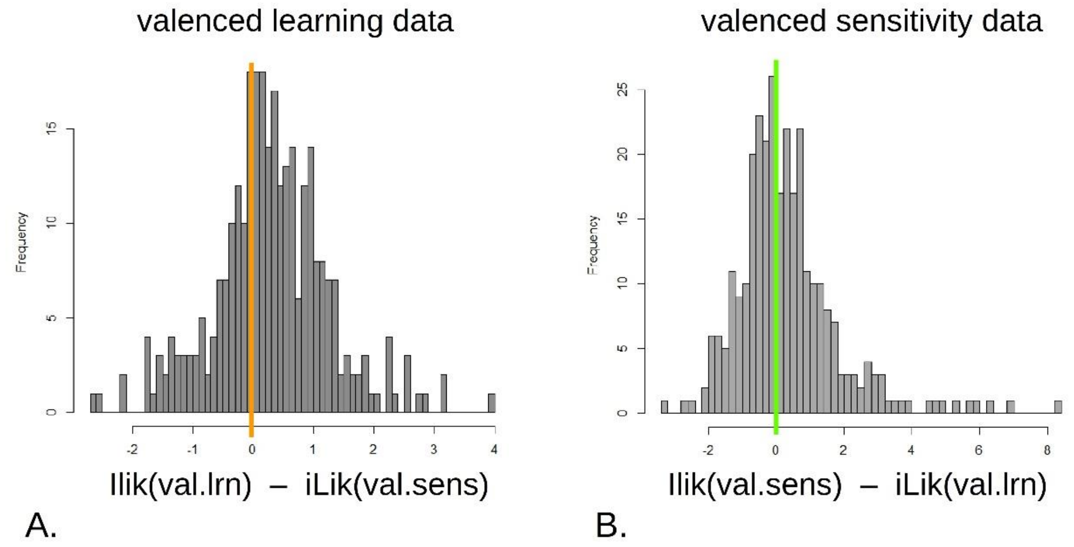

Fig S9. Confusion analyses between the two best models. **A.** Distribution of fitted integrated likelihood differences (here identical to iBIC differences) when the true generative model is the two-learning-rate (val.lrn) one. 35% of datasets lie below zero (gold line). **B.** Similar for data generated with the two-sensitivity model (val.sens). 47% of datasets lie to left of the green dividing line.

We also checked for biases that the fitting procedure could induced, which could, in principle, influence our interpretation of analogous findings in the estimation of parameters from real data. The worst case of bias took place when generative Pavlovian parameters were very low. Re-fitting inflated these values, although its impact in describing variance within the group was low (Fig S8A and S9).

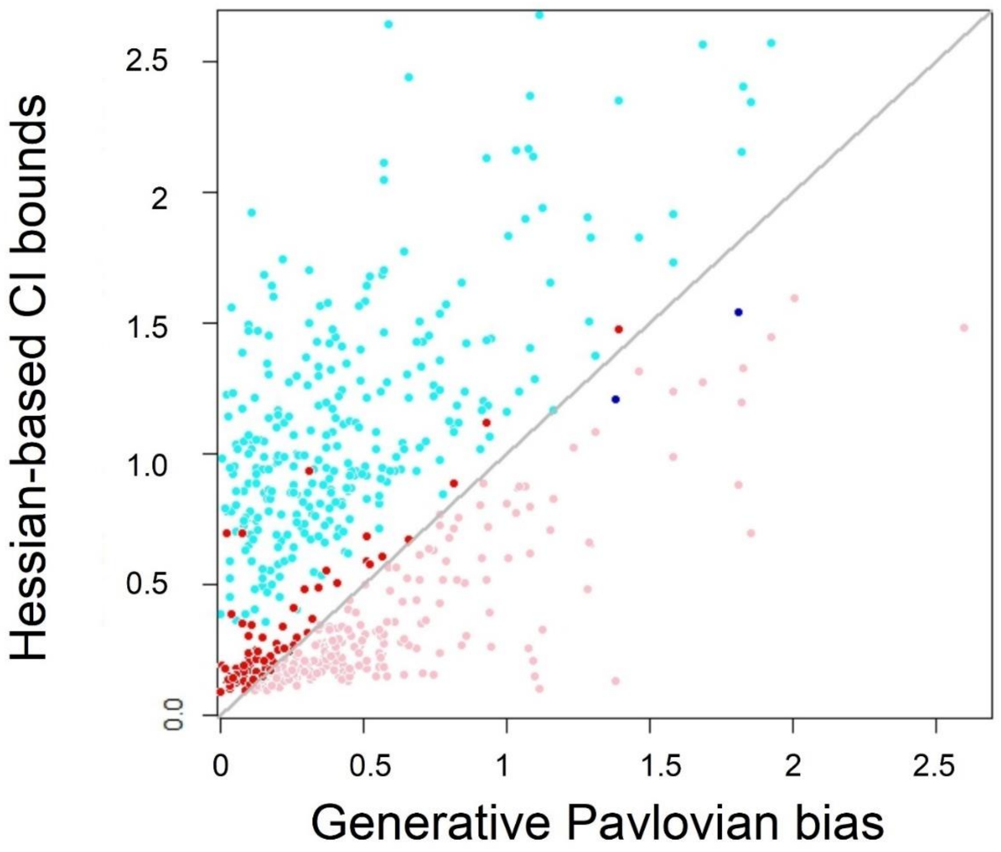

Fig. S10. Estimation bias in fitted values of the Pavlovian bias parameter, for a similar analysis as in fig. 5a in the main text, but using the valenced-sensitivity model with generative parameters resampled from the ones fitted to the baseline sample. Here we plot the lower bounds (LB) of the 95% confidence intervals estimated by the fitting algorithm in pink or red, and the upper ones in blue or cyan (UB). Despite the best-estimate fitted values being 0.84 correlated with the generative ones, the fitting routine overestimates its own accuracy so that 30% of intervals have either an UB below the diagonal (blue rather than cyan) or, much more commonly, a LB above the identity line (red rather than pink). This is the worst case we encountered in simulations.

Finally, we checked the propensity of re-fitting to induce spurious correlations between fitted parameters, to help interpret table 1. Using uncorrelated generative parameters within the range of our baseline sample, the appetitive sensitivity of the re-fitted valenced-sensitivity model was

spuriously correlated positively both with learning rate (spearman ρ = 0.24, corrected p= 4.8e-4) and with Pavlovian bias (spearman ρ = 0.22, corrected p= 0.0026). However, no spurious correlations was observed between Pavlovian bias and learning rates, which means that the associations between them found in the real data are likely to be a true feature of the study population rather than a modelling artefact.

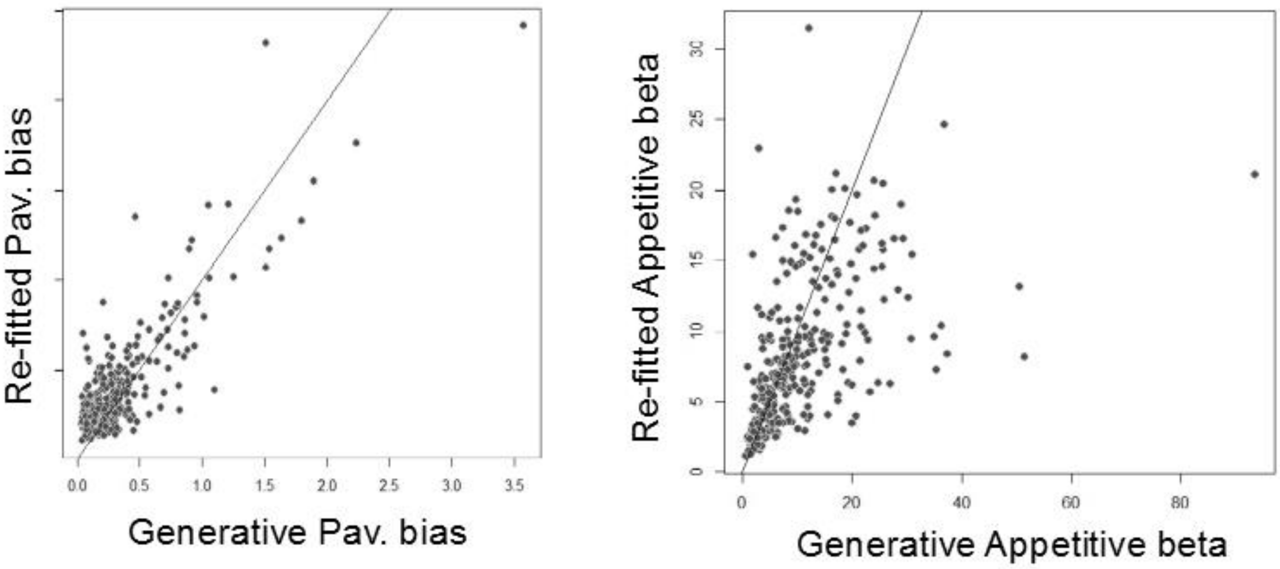

a. b.

Fig S11. Simulated, then re-fitted parameters based on the valenced sensitivity model without forgetting, using group-level generative parameters derived from the fit to the baseline sample, similar to Fig. 1. In each plot the straight line is the identity map. **a.** Pavlovian Bias is retrieved quite well overall. r=0.85, p < 1e-05 **b.** Motivational exchange rate, here for the win trials, is retrieved somewhat less well (r=0.54, p < 1e-05 including outlier).

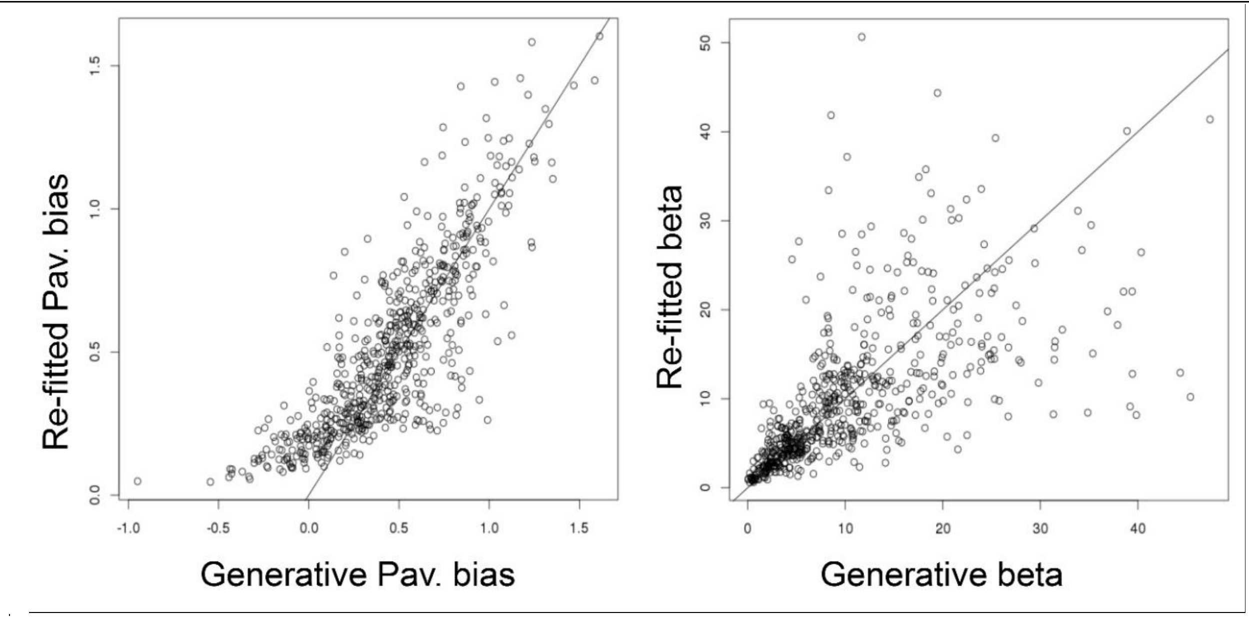

a. b.

Fig S12. Simulated, then re-fitted parameters based on the valenced learning model without forgetting. The mean and variance of the fit of the model to the short-follow-up study data was used to generate plausible simulated data. However an added twist was added, in that the generative distribution for Pavlovian bias was allowed to have a negative tail to explore the effect that the assumed shape of the fitted group distribution may not quite match the (unknown) real one. In each plot the straight line is the identity map. **a.** Pavlovian Bias is retrieved well overall, despite the formal inability to fit putative negative biases. r=0.83, p < 1e-05 **b.** Motivational exchange rate is again retrieved less powerfully, as in Fig. 1 of the main text. r=0.64, p < 1e-05.

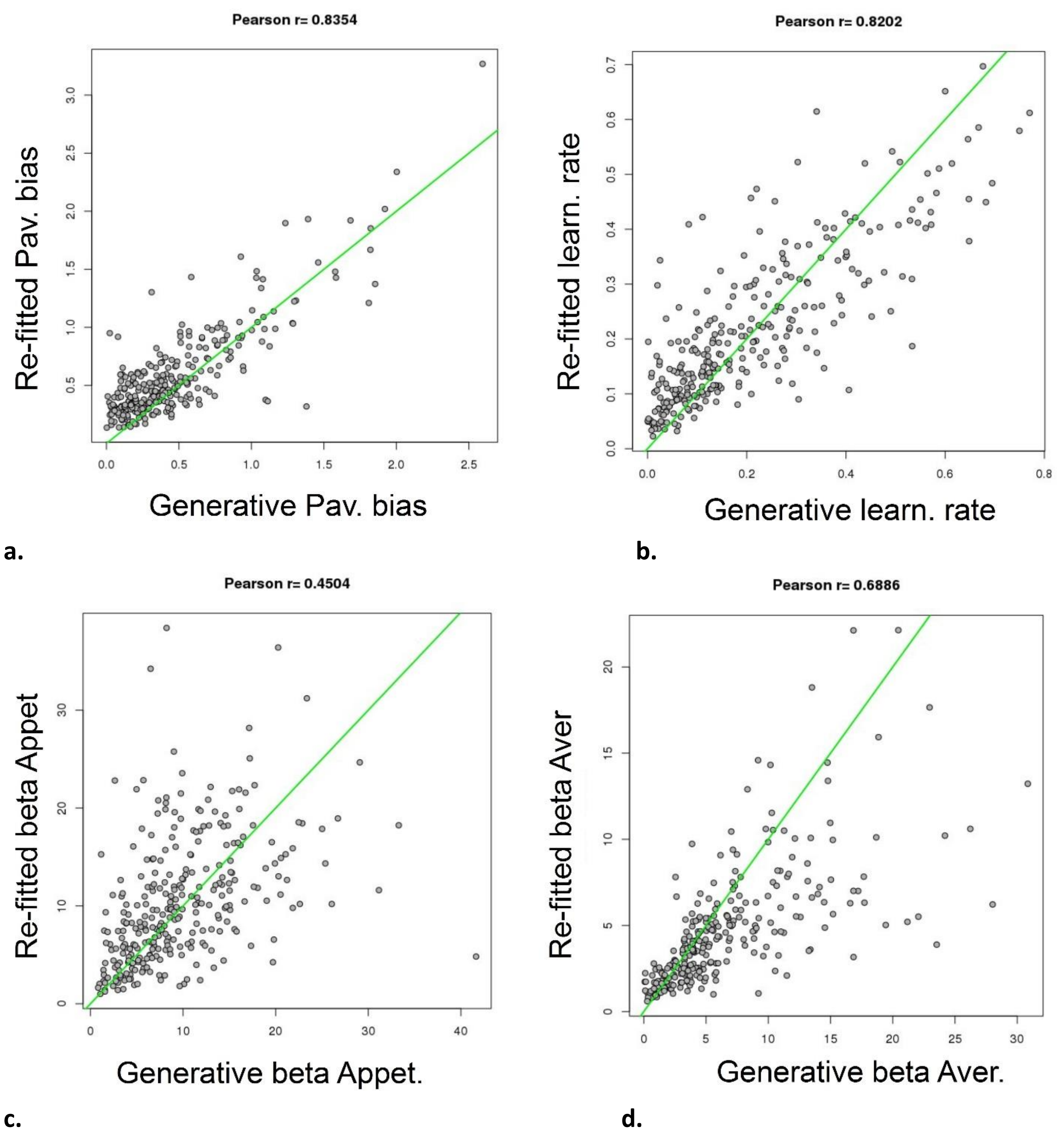

Fig S13. Simulated, then re-fitted parameters based on the valenced sensitivity model without forgetting. The means and variances of the population distributions are as per Fig. 4, but here gamma population distributions are used for the Pavlovian and sensitivity parameters, and beta for the learning rate. Parameters are retrieved as well as when the formally correct distributions are used. **a.** Pavlovian Bias **b.** Learning rate **c.** Appetitive sensitivity. This is the least well recovered here. **d.** Aversive sensitivity.
