# Supplementary material for: Change, stability, and instability in the Pavlovian guidance of behaviour from adolescence to young adulthood
Source: PLoS Comput Biol. 2018 Dec 31;14(12):e1006679. doi: 10.1371/journal.pcbi.1006679 (PMC6329529; doi:10.1371/journal.pcbi.1006679)
Supplement: S2 Appendix — (PDF) [file pcbi.1006679.s008.pdf]

Appendix S8: Clustering analyses

We performed clustering analyses using the MClust package in R (Scrucca, Fop, Murphy, & Raftery, 2017).

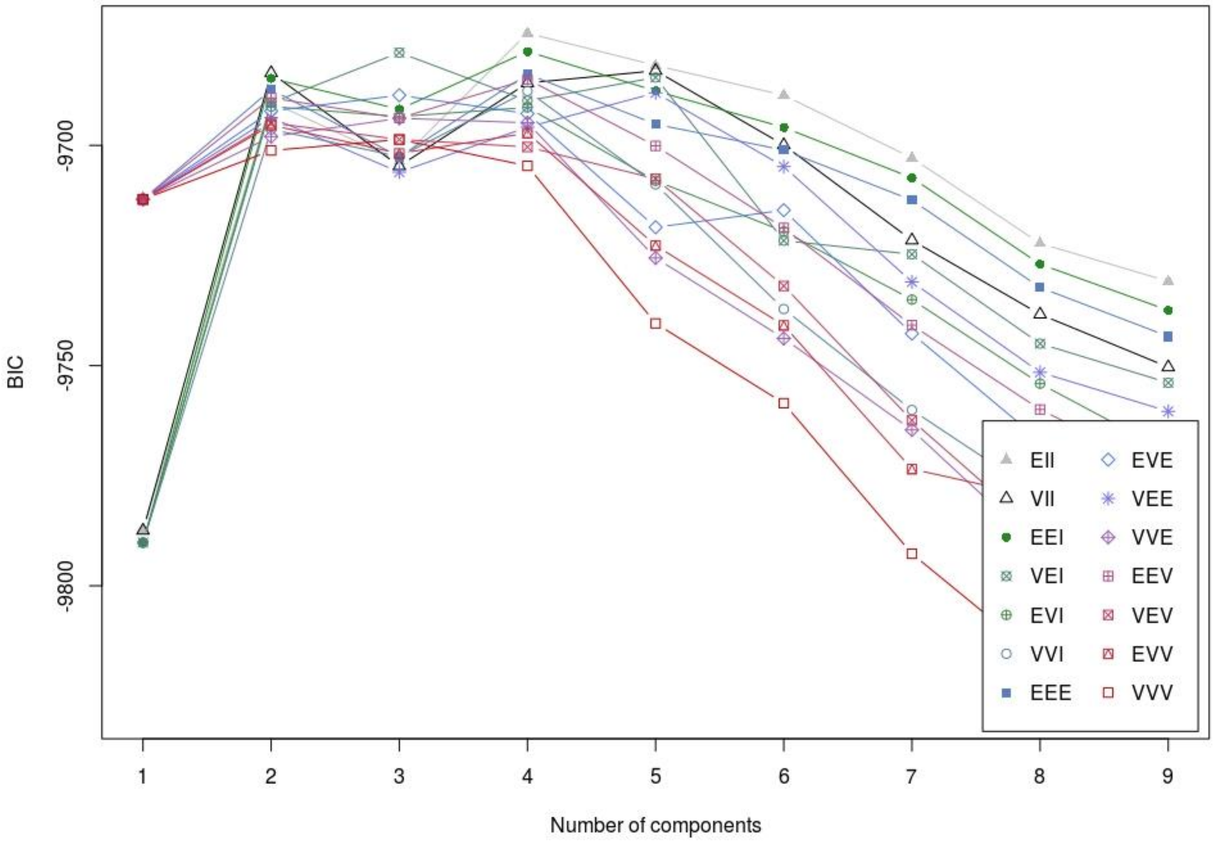

Fig. S15 BIC plot for model comparison among a large variety of clustering solutions to describe the joint distribution of baseline and long follow-up model-fit (integrated likelihood) measures. The spherical, equal-volume Gaussian model resulting in the four-cluster solution discussed in the main text is the grey triangle 'EII' at 4 components. Key: "EII" : spherical, equal volume; "VII" : spherical, unequal volume; "EEI" : diagonal, equal volume and shape; "VEI": diagonal, varying volume, equal shape ; "EVI" : diagonal, equal volume, varying shape; "VVI" : diagonal, varying volume and shape; "EEE" : ellipsoidal, equal volume, shape, and orientation; "EEV" : ellipsoidal, equal volume and equal shape "VEV": ellipsoidal, equal shape "VVV": ellipsoidal, varying volume, shape, and orientation.

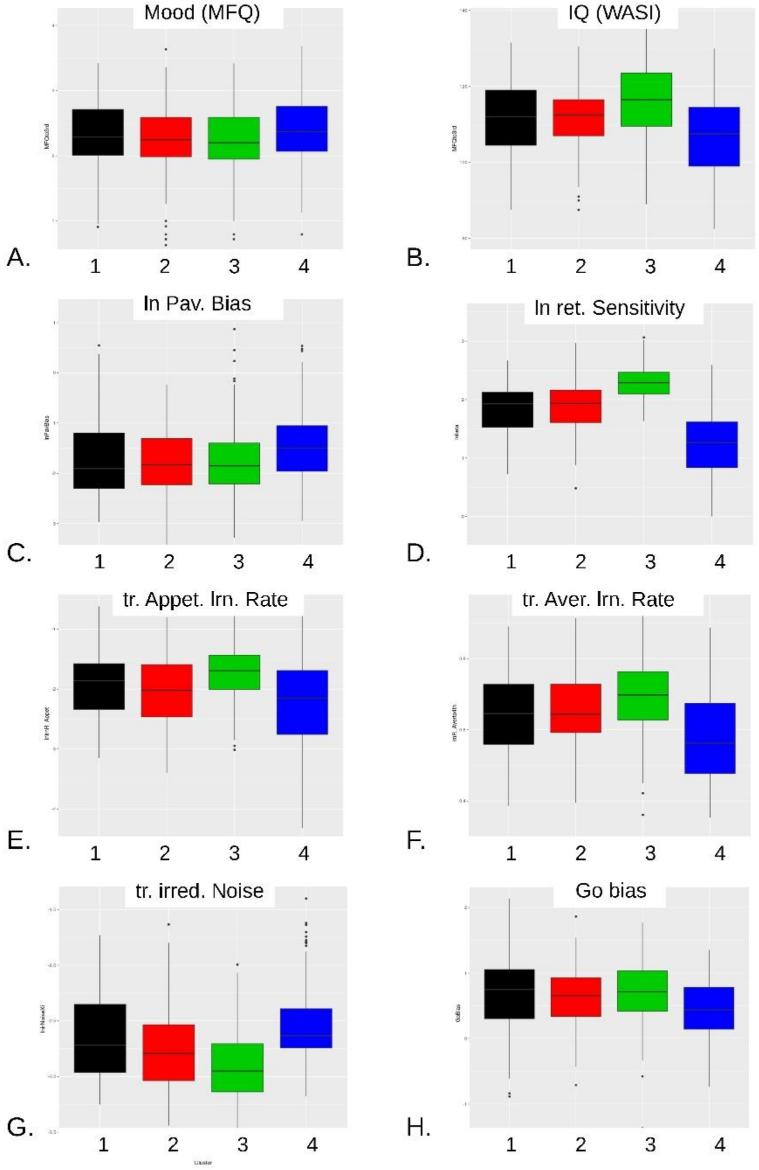

Fig S16. Simplified plots showing Mood, IQ and parameter values for the four clusters identified. Y axes are transformed / rescaled to facilitate visual comparisons. There are no significant differences in Mood (A.). The rest of the plots (B.-H.) show very similar values for the two smaller clusters, 1 and 2 but the blue cluster, 4, who had the best fits in both baseline and long follow-up, consistently has parameters more advantageous to better performance, and higher IQ, whereas the green cluster, 3, shows the exact opposite pattern. See table below for formal statistical comparisons.

The table below shows post-hoc contrast for differences between means of parameters, Mood and IQ of the four different clusters.

```
Mood (MFQ to power 1/3)
           z value  Pr(>|z|)
2 - 1 == 0 -1.717   0.5162
3 - 1 == 0 -1.421   0.9321
4 - 1 == 0  0.483   1
3 - 2 == 0  0.480   1
4 - 2 == 0  2.492   0.0762
4 - 3 == 0  2.269   0.1397
```

```
IQ
           z value  Pr(>|z|)
2 - 1 == 0   0.537         1
3 - 1 == 0   4.883  6.27e-06 ***
4 - 1 == 0  -5.165  1.44e-06 ***
3 - 2 == 0   4.578  2.82e-05 ***
4 - 2 == 0  -6.115  5.79e-09 ***
4 - 3 == 0 -11.997   < 2e-16 ***
```

```
Pavlovian Bias (log transf.)
           z value Pr(>|z|)
2 - 1 == 0  -0.968 1
3 - 1 == 0  -0.762 1
4 - 1 == 0   2.682 0.043866 *
3 - 2 == 0   0.313 1
4 - 2 == 0   3.970 0.000431 ***
4 - 3 == 0   4.119 0.000228 ***
```

```
Return Sensitivity (log transf.)
           z value Pr(>|z|)
2 - 1 == 0  0.814        1
3 - 1 == 0  8.022  6.66e-15 ***
4 - 1 == 0 -10.019  < 2e-16 ***
3 - 2 == 0  7.599  1.79e-13 ***
4 - 2 == 0 -11.600  < 2e-16 ***
4 - 3 == 0 -21.547  < 2e-16 ***
```

```
Appet. Lrn. rate (log transf.)
           z value Pr(>|z|)
2 - 1 == 0 -0.440  1
3 - 1 == 0  2.936  0.01995 *
4 - 1 == 0 -3.270  0.00646 **
3 - 2 == 0  3.638  0.00165 **
4 - 2 == 0 -2.973  0.01770 *
4 - 3 == 0 -7.410 7.57e-13 ***
```

```
Aver. Lrn. rate (raised to 1/4)
           z value Pr(>|z|)
2 - 1 == 0  0.782  1
3 - 1 == 0  3.583  0.002040 **
4 - 1 == 0 -4.263  0.000121 ***
3 - 2 == 0  2.908  0.021816 *
4 - 2 == 0 -5.437  3.24e-07 ***
4 - 3 == 0 -9.369   < 2e-16 ***
```

```
Irreducible noise (log transf.)
           z value Pr(>|z|)
2 - 1 == 0 -1.343  1
3 - 1 == 0 -5.276  7.93e-07 ***
4 - 1 == 0  2.989  0.016774 *
3 - 2 == 0 -4.060  0.000295 ***
4 - 2 == 0  4.729  1.35e-05 ***
4 - 3 == 0  9.858   < 2e-16 ***
```

```
Go bias
           z value Pr(>|z|)
2 - 1 == 0 -0.846  1
3 - 1 == 0  0.903  1
4 - 1 == 0 -3.510  0.00269 **
3 - 2 == 0  1.944  0.31164
4 - 2 == 0 -2.761  0.03463 *
4 - 3 == 0 -5.278  7.82e-07 ***
```

Table S17. Tukey contrasts for multiple comparisons of means of the four clusters. Note how group 4, that fitted poorly on at both baseline and long follow-up stands out as different to the others and starkly contrasts with group 3, which fitted best on both occasions. Tukey tests were done separately for each measure, following linear mixed effects analyses. All entire analyses are statistically significant, at least $p < 0.0001$, except for Mood which is non-significant. A rigorous criterion would be to threshold the values above for 0.05/8 = 0.00625, and 0.01/8=0.00125, which means that in the table '*' = trend, '**' = stat. sig. at 0.05, '***' = stat. sig. at least 0.01. Note that the significant results regarding return sensitivity and irreducible noise look strong but are not independent from the measure that was used to cluster participants, and hence offer little new information.

If a clustering solution is forced to utilize two clusters only, corresponding to the two clear peaks of the distribution, two equipopulous clusters form, the first, C1, approximately corresponding to the union of the green and red (2 and 3) clusters above, and C2 corresponding to the union of the black and blue (1 and 4) clusters. The parameter and likelihood comparison between these two larger clusters is shown in Table S18.

	motiv. exchange rate	appet. lrn	aversive lrn.	pavlovian bias	irred. noise	Go bias	int.likelihood (predict. per trial)
Baseline							
mean C1	6.64	0.16	0.20	0.46	0.087	0.61	-74.2 (59.7%)
mean C2	9.26	0.18	0.27	0.35	0.079	0.74	-59.5 (66.1%)
Pcorr.	<1e-10	0.010	9.1e-6	0.56	0.011	ns	<1e-10
Long Follow-up							
mean C1	5.13	0.20	0.22	0.30	0.085	0.46	-80.6 (57.1%)
mean C2	10.85	0.22	0.30	0.23	0.054	0.66	-45.8 (72.7%)
Pcorr	p<1e-10	0.0012	<1e-10	0.34	<1e-10	0.015	<1e-10

Table S18. Means of the parameters if participants are classified two clusters, C1 and C2, of the joint distribution over integrated likelihood. P values regard Wilcoxon comparisons between clusters at the same time point, corrected for 14 comparisons. Note that the model accounts significantly better for the behaviour of the second cluster at both timepoints (better int. likelihood / predictability per trial).
