# Supplementary material for: Change, stability, and instability in the Pavlovian guidance of behaviour from adolescence to young adulthood
Source: PLoS Comput Biol. 2018 Dec 31;14(12):e1006679. doi: 10.1371/journal.pcbi.1006679 (PMC6329529; doi:10.1371/journal.pcbi.1006679)
Supplement: S3 Appendix — (PDF) [file pcbi.1006679.s009.pdf]

Appendix S9: Latent Change Score Modelling

Here we illustrate the general structure of LCSMs that we used, and we describe an important control analysis, namely whether changes in parameters may have differed depending on baseline age or IQ.

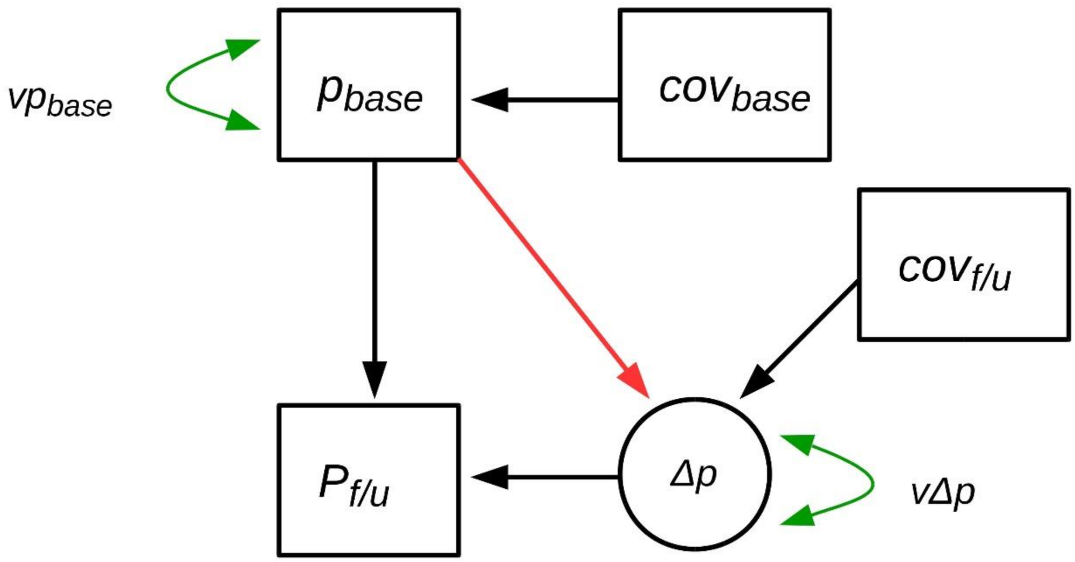

Fig S20. Univariate latent change score model for a parameter estimated at baseline (p_{base}) and follow-up ($p_{f/u}$). The change is conceptualized as a latent factor *Δp* which absorbs the variance observed in the difference between the two measurements. Therefore the regression weights of p_{base} and Δp upon $p_{f/u}$ are fixed at 1. In addition, covariates such as exact age difference between the two measurements, or average IQ over the duration of the experiment, can be used to test whether either the overall level or the change of the score of interest depends on the covariate. The intercepts and variances of $p_{f/u}$ and Δp as well as the dependence of the regression weight $\Delta p \sim p_{f/u}$ on p_{base} is estimated from the data. Squares: Observed data. Circles: latent variables. Single-headed arrows: regressions (only the red is estimated from data). Double headed arrows: variances and covariances.

In order to see whether age and IQ may have accounted for changes in Pavlovian bias, we augmented the latent change score model (LCSM) with an age (mean over the two testing occasions) and baseline IQ, which were entered as covariates in the latent change score. We found no dependence of the change in Pavlovian bias on age (regression p=0.75 within the LCSM) but there was a small, significant dependence of change in Pavlovian bias on IQ, p=0.028, beta=-0.008, r^2 = 0.005. This means that the higher the IQ, the change in Pavlovian bias was slightly smaller/more negative than would be predicted on the basis of the other predictors in the LCSM, but this only accounted for 0.5% of the variance in change.
