# Supplementary material for: Change, stability, and instability in the Pavlovian guidance of behaviour from adolescence to young adulthood
Source: PLoS Comput Biol. 2018 Dec 31;14(12):e1006679. doi: 10.1371/journal.pcbi.1006679 (PMC6329529; doi:10.1371/journal.pcbi.1006679)
Supplement: S1 Table — (PDF) [file pcbi.1006679.s014.pdf]

Neuroscience in Psychiatry Network Study & Consortium Author list	
Principal Investigators	Edward Bullmore (CI from 01/01/17)
	Ian Goodyer (CI until 01/01/2017)
	Raymond Dolan
	Peter Fonagy
	Peter Jones
NSPN funded staff	Michael Moutoussis
	Tobias Hauser
	Petra Vértes
	Kirstie Whitaker
	Gita Prabhu
	Laura Villis
	Junaid Bhatti
	Becky Inkster
	Cinly Ooi
	Barry Widmer
	Ayesha Alrumaithi
	Sarah Birt
	Kalia Cleridou
	Hina Dadabhoy
	Sian Granville
	Elizabeth Harding
	Alexandra Hopkins
	Daniel Isaacs
	Janchai King
	Danae Kokorikou
	Harriet Mills
	Ciara O'Donnell
	Sara Pantaleone
Affiliated Scientists	Pasco Fearon
	Anne-Laura van Harmelen
	Rogier Kievit

Table S1. Neuroscience in Psychiatry consortium author list.
