# Supplementary material for: Change, stability, and instability in the Pavlovian guidance of behaviour from adolescence to young adulthood
Source: PLoS Comput Biol. 2018 Dec 31;14(12):e1006679. doi: 10.1371/journal.pcbi.1006679 (PMC6329529; doi:10.1371/journal.pcbi.1006679)
Supplement: S2 Table — Correlation values below the diagonal, corrected p values above. Note the absence of significant correlation between appetitive and aversive sensitivity, in line with the worse fitting of the sensitivity-ratio model compared to the valenced-sensitivity one. (PDF) [file pcbi.1006679.s015.pdf]

	bet_Appet	bet_Aver	lrnRate	PavBias	irr. Noise	GoBias
bet_Appet	-	ns	ns	ns	ns	***p*=0.002**
bet_Aver		-	ns	ns	***p*< 1e-10**	ns
lrnRate			-	***p*< 1e-10**	ns	ns
PavBias			***r*= -0.27**	-	ns	ns
irr. Noise		***r*= -0.268**			-	ns
GoBias	***r*= 0.137**					-

Table S2. Posterior spearman correlations at the 0.01 level, corrected for 6*5/2 comparisons, for the baseline sample, for the valenced sensitivity model. Correlation values below the diagonal, corrected *p* values above. Note the absence of significant correlation between appetitive and aversive sensitivity, in line with the worse fitting of the sensitivity-ratio model compared to the valenced-sensitivity one.
