# Supplementary material for: Change, stability, and instability in the Pavlovian guidance of behaviour from adolescence to young adulthood
Source: PLoS Comput Biol. 2018 Dec 31;14(12):e1006679. doi: 10.1371/journal.pcbi.1006679 (PMC6329529; doi:10.1371/journal.pcbi.1006679)
Supplement: S3 Table — Other things being equal, a decrease in Pavlovian bias should improve performance in the Pavlovian-inconsistent conditions (+ve pearson r by convention in this table) and decrease performance in the Pavlovian-consistent conditions (-ve r). To test this, we tested the hypothesis on each condition separately, adopting a multiple comparison threshold of p = 0.05/4 = 0.0125. We found significant evidence for all expected correlations. (PDF) [file pcbi.1006679.s016.pdf]

Measure	Pearson *r*	P value
Change in Pavlovian – Congruent conditions:		
r(change in G2W performance , reduction of Pav. Bias)	-0.11	0.010
r(change in NG2AL performance , reduction of Pav. Bias)	-0.20	1.7e-06
Change in Pavlovian – Incongruent conditions:		
r(change in NG2W performance , reduction of Pav. Bias)	0.33	< 1e-10
r(change in G2AP performance , reduction of Pav. Bias)	0.62	< 1e-10

Table S3. Support for the theoretically expected relationship between Pavlovian bias and performance. Other things being equal, a decrease in Pavlovian bias should improve performance in the Pavlovian-inconsistent conditions (+ve pearson r by convention in this table) and decrease performance in the Pavlovian-consistent conditions (-ve r). To test this, we tested the hypothesis on each condition separately, adopting a multiple comparison threshold of $p=0.05/4 = 0.0125$. We found significant evidence for all expected correlations.
